# Supplementary material for: Predicting targeted- and immunotherapeutic response outcomes in melanoma with single-cell Raman spectroscopy and AI
Source: bioRxiv. 2026 Mar 12:2025.05.16.654612. Preprint. [Version 2] doi: 10.1101/2025.05.16.654612 (PMC13014145; doi:10.1101/2025.05.16.654612)
Supplement: Supplement 1 [file media-1.pdf]

Supplementary Methods: Experimental

Melanoma cell lines

Cells were cultured in complete cell culture growth medium including RPMI (Gibco, Grand Island, NY) supplemented with 10% fetal bovine serum (Corning, Woodland, CA) at 37°C in an incubator supplied with 5% CO2. Media was changed at least twice a week. Cells were cultured at 37°C in a humidified CO2 incubator at 5% CO2. Cell lines not used in drug response were cultured until 65-70% confluency. Cell lines used in drug response were cultured to 75-80% confluency, trypsinized (Trypsin-EDTA, Gibco) for 5 min at 37°C, collected in suspension in a 15 mL tube, and centrifuged at 400 g for 5 minutes at 4°C. The supernatant was removed, and the cell pellet was resuspended with 1 mL RPMI complete media. Cells were counted and plated at a density of 1x106 cells/well into a 6-well plate and placed overnight in an incubator for adherency. Cells were then treated with desired inhibitors with varying concentrations for 24 hours. Inhibitor concentrations for all samples can be found in Supplementary Table 2. Duplicate wells were used for both treated and untreated cells. Plates were incubated for 24 hours. Dead cells along with the drug media were removed, and cells were trypsinized for 5 minutes, collected into 15 mL tubes, and centrifuged at 400 g for 5 minutes. After supernatant removal, cells were resuspended into 1 mL of media, and cell viability was assessed with Trypan blue assay. Hits were identified using a threshold of 65-70% inhibition of cell viability. For temporal studies, cells from the same batch were split equally and treated with bemcentinib for 30 minutes or 24 hours prior to Raman analysis.

Human melanoma patient-derived cells

Melanoma tissues were collected in RPMI media and transported to lab on ice and rinsed with DPBS (Gibco) and removal of excess connective and vascular tissues. Subsequently, tissues were finely minced using a sterile scalpel and collected in the tube. After centrifugation (5 min at 4°C, 400 g) the cell pellet was resuspended in DMEM/F12 (Invitrogen, Waltham, MA), 12 mM HEPES (Invitrogen), 1% GlutaMAX (Invitrogen), 10% fetal bovine serum (FBS), supplemented with 9 µM ROCK (Y-27632) inhibitor (Selleckchem, Houston, TX) and 1% penicillin/streptomycin (Invitrogen). Cells were seeded in a cell culture dish and kept at 37°C and 5% CO2 in an incubator. Once they reached 65-70% confluence, cells were detached with TrypLE Express (Gibco), centrifuged (5 min at 4°C, 400 g) and seeded in 6 well plates at a density of 1x106 cells per well for drug treatment. For select patient samples, persistent cells were further partitioned for fixation protocols used in Raman data acquisition and for preparation for single-cell transcriptomic sequencing.

scRNA-seq cell and library preparation

Patient-derived melanoma cell lines were trypsinized for 5 minutes, collected into 15 mL tubes, then spun down at 400 g for 5 min at 4°C. After removing the supernatant, the cells were resuspended in 1 mL of PBS with 0.5% BSA. Live and dead cells were visualized and counted using Trypan blue. If over 40% of dead cells were present, the sample was spun down at 400 g for 5 min at 4°C and subject to the dead cell removal kit (Miltenyi Biotec, 130-090-101). Finally the samples were spun down at 400 g for 5 min at 4°C and resuspended in 500 µL - 1 mL of PBS for a final concentration of 1000-1,500 cells/µL for a target cell recovery of 20,000 cells. For the

characterization studies, library preparation was performed using Chromium GEM-X Single Cell 3' Reagent Kits v4 (10x Genomics). Library conversion was then done using Singular Genomics Library Compatibility Kit (Singular Genomics). Libraries were sequenced on the Singular Genomics G4 sequencer, using 100 cycle F3 flow cells (Singular Genomics), with two lanes per library at 28/90 paired end.

Intracellular neutral lipid staining of live cells

Approximately 10^5 YUMMER1.7 cells per sample were harvested and washed with FACS buffer, PBS (20012-050, Gibco) + 0.2% BSA (A9418, Sigma), twice. Cells were stained with 2 μM BODIPY 493/503 (D3922, Thermo) for 30 min at 37°C. Cells were again washed twice with FACS buffer and then resuspended in 1 μg/mL DAPI (D1306, Thermo). Mean fluorescence intensity (MFI) of BODIPY signal was quantified on a CytoFLEX S Flow Cytometer (Beckman Coulter) by gating on live (DAPI-) single cells. Comparison of multiple groups was performed by using one-way analysis of variance (ANOVA) with Dunnett correction.

Intracellular protein and phosphorylation quantification

$6x10^6$ YUMMER1.7 cells per sample were lysed on ice for 45 minutes with RIPA Lysis and Extraction Buffer (8990, Thermo) supplemented with Vanadate (P0758L, New England Biolabs), Halt Protease Inhibitor Cocktail (#78430, Thermo), Phosphatase Inhibitor Cocktail I (AB201112, Abcam), and Protease Inhibitor Cocktail (P8849, Sigma), and DDM (D310S, Calibre Scientific). Samples were divided for use in total intracellular protein and phosphorylation quantification. Total intracellular protein quantification was performed using the Micro BCA Protein Assay Kit (23235, Thermo) according to manufacturer instructions. Sample protein concentrations were interpolated from a standard curve using GraphPad Prism (v. 10.6.0). Comparison of multiple groups was performed by using one-way analysis of variance (ANOVA) with Dunnett correction. Phosphorylation quantification was performed via Western Blot by detecting phosphorylated serine and threonine residues with tyrosine, tryptophan, or phenylalanine at the -1 position or phenylalanine at the +1 position. Samples were resolved by SDS-PAGE, with an equal proportion of cell lysate from each sample loaded in each lane. Phosphorylation levels were analyzed by immunoblotting using a 1:1000 dilution of Phospho-(Ser/Thr) Phe Antibody (9631, Cell Signaling Technology) for primary staining and a 1:10000 dilution of IRDye 680RD Goat Anti-Rabbit IgG (926-68071, LICORbio). The blotted membrane was imaged using a LICOR Odyssey imaging system. Signal intensity was quantified using ImageJ 1.54 and plotted in GraphPad Prism (v. 10.6.0). Comparison of multiple groups was performed by using one-way analysis of variance (ANOVA) with Dunnett correction.

Cell fixation for Raman spectroscopy

Upon confluency, media was aspirated and the cells were washed with 5 mL of PBS. After removal of media, 3 mL of 0.25% trypsin-EDTA was added to the culture flask and incubated at 37°C at 5% CO2 for 5-7 min. After incubation, RPMI + 10% FBS was added to quench the trypsin reaction, and the cell suspension was collected into a 15 mL centrifuge tube. The tube was spun for 5 minutes at 400 g, and the supernatant was aspirated. Next, 1 mL of 4% paraformaldehyde was added, and the solution was mixed gently and incubated at room temperature for 10 minutes. After incubation, the tube was centrifuged for 3 minutes at 400 g at 4oC before supernatant

removal. The cell pellet was resuspended with an additional 1 mL PBS and centrifuged for 3 minutes at 400 g. The supernatant was aspirated, 50 uL of PBS was added, and the solution was gently mixed. Based on cell concentration, 1-5 uL of cell solution was drop casted on a gold-coated slide (~195 nm Au, 5 nm Ti, 1 mm glass) and air dried for 5-10 minutes prior to Raman acquisition. For live cell preparation, cells were removed prior to paraformaldehyde and suspended in 50 uL of PBS.

Targeted inhibitors for Raman spectroscopy

Powders of high purity (>99.9%) bemcentinib, cabozantinib, and dabrafenib were each thoroughly mixed with 3 uL of PBS with a pipette. 2 uL of mixture was dropcast on a gold-coated slide and air dried for 5-10 minutes prior to Raman acquisition.

Raman data acquisition

Raman spectra on the dried samples were acquired using a confocal Raman spectrometer (Horiba LabRAM, Longjumeau, France) at 532 nm excitation with a 100x 0.6 NA objective at five second integration times. Using a 600 gr/mm grating, the spectral acquisition range acquired was 500-2000 cm^{-1}. All spectra, unless otherwise stated, were acquired with 100% power output of ~5.4 mW with a nominal spot size of ~2.1 µm. Spectra for murine sample B16-F10 and human samples SK-MEL-30 (control) and PAT-73 (bemcentinib) were collected at both 100% and 50% power using an integrated ND filter. Spectra for murine samples YUMM (cabozantinib, dabrafenib, nivolumab) were collected at both 25% and 50% power. Spectra were acquired using two approaches: a) via a rectangular grid mapping with 20 µm spacings between point measurements for throughput, and b) via targeted point-by-point selection per window region on visually identified cells to ensure approximate single-cell acquisition and minimize cell measurement duplicates. While the confocal configuration provided subcellular lateral resolution, finite axial depth of field combined with occasional 2-3 cell layering meant some grid-mapped spectra contained contributions from multiple cells. Point-by-point measurements on isolated cells ensured single-cell data. Spectra for targeted inhibitors were acquired with 25% power for dabrafenib and bemcentinib and at 100% power for cabozantinib. In total, 8,095 cell spectra were utilized for murine TIME cell differentiation, 3,594 for macrophage polarization, 9,937 for murine drug response, 33,921 for human drug response, and 23,994 for melanoma resistance determination. For biochemical validation, 2,188 cell spectra were utilized.

Raman data acquisition for live vs fixed comparison

Raman spectra on fixed cells and live cells at 532 nm excitation followed the preparation and measurement protocols described prior. Live cells suspended in PBS medium were placed in a liquid well constructed of a gold-coated glass slide base layer with hole-punched, double sided adhesive tape serving as the side walls. Vacuum grease was utilized on the edge of the double sided tape to prevent sample leakage, and the well was covered with a borosilicate glass cover slip to prevent sample evaporation. 4 µL of cell solution was placed in the liquid well. Spectra at 785 nm excitation was acquired using a confocal Raman spectrometer (Horiba XploRA+, Longjumeau, France) using a 50x/0.65 NA objective (Olympus LCPLAN 50X IR) with a 0.5 correction collar. Using a 1200 gr/mm grating, the spectral acquisition range acquired was 500-2000 cm^{-1}. All spectra were acquired with 100% power output of 26.34 mW with a nominal spot

size of ~1.5 µm. Spectra were acquired at 20 second integration times. The 50x objective was additionally used for sequential time series acquisitions to minimize background contribution from the cover slip.

Data preprocessing

Spectra were first filtered by an intensity count threshold to remove detector saturated measurements. Unprocessed Raman spectra were then despiked using a modified Whitaker-Hayes method to remove cosmic rays and spiked pixel regions on the CCD[1]. Upon despiking, spectra were denoised via wavelet thresholding. The python package skimage was used to perform our smoothing, and BayesShrink was chosen as our wavelet coefficient threshold selection method[2]. Following our spectral smoothing, a baseline correction was applied using a combination of polynomial fitting (4th order) and adaptive iteratively reweighted Penalized Least Squares (airPLS)[3]. The correction method was implemented using the python packages PeakUtils and BaselineRemoval[4,5]. Each baseline-subtracted spectrum was internally normalized by replacing the intensity of each wavenumber with the standard score, calculated from the mean and standard deviation of the intensities in the spectrum, to enable cross-spectrum comparisons. The biological fingerprint region of 600-1800 cm^{-1} of the processed spectra was isolated for analysis. Off-cell measurements from grid mapping, stage translation drifts, and background media and poor-quality spectra characterized by high background to cell signal intensities were filtered utilizing a combination of one-class support vector machines and major biological bands z-scores for all datasets. The set of one-class support vector machines (SVMs, sklearn)[6] was trained using spectra from relevant backgrounds (gold slide, phosphate-buffered saline, supernatant, polystyrene).

Supplementary Methods: Data Analysis

Spectral clustering

Preprocessed data were subjected to linear dimensionality reduction by PCA (sklearn) to reduce the feature size to a set of principal components explaining ≥95% of the dataset variance. The transformed dataset aided in visualization and in certain cases, improved classification runtimes. Manifold learning (UMAP, umap and Scanpy)[7,8] was applied to the PCA-reduced dataset to visualize the samples in a two-dimensional embedding, and hyperparameters random_seed=42, n_neighbors=25, min_dist=0.5, spread=1, n_epoch=500 were used across all datasets, with adjustments of the following: min_dist=0.3, spread=2 for murine cell differentiation described in Figure 2b; n_neighbors=30 for mice drug response, min_dist=0.3 for human drug response, and min_dist=0.3, spread=3 for patient data. Effects of hyperparameters on UMAP embedding are highlighted in Supplementary Figure 17. Leiden clustering[9] (Scanpy) with resolution 0.3 (human response) and 0.5 (patient dataset) was used to examine spectral differences among clusters. Heatmap (differential Raman) analysis was performed by first shifting our dataset by a constant c, where c is the value needed to raise our minimum value to nonzero. After, the median value of the wavenumber features were determined from our control group. Median was selected over arithmetic mean for improved robustness against dissimilar group distributions[10]. In the case of the drug screening assay, the untreated cells served as control. Finally the fold change was determined for each sample through the log2 of the ratio between sample features and the control group. Log2 was chosen such that a two fold increase (doubling of quantity) resulted in a log2 fold change of +1 whereas a two fold decrease (halving of quantity) resulted in a log2 fold change of -1. Wavenumber selections for the differential intensity heatmaps were either determined from the top wavenumbers affecting classification accuracy by perturbation calculations (see feature importance section below) or by the combination of most prominent peak selection in the average spectra (SciPy)[11] and highest variant wavenumbers using Wilcoxon rank-sum (Scanpy). Cell spectra were subsampled for heatmap visualization.

Spectral barcodes

Barcodes using the top 50 variant wavenumbers were generated from averaging the log2 fold change of 500 randomly selected spectra from each sample. Cell spectra with predicted probabilities of >0.8 and <0.2 from each of the drug determination classifiers were selected for high-likelihood resistant and sensitive barcodes, respectively. Barcodes were color bound symmetrically with a ±0.5 for bemcentinib and cabozantinib, ±0.75 for nivolumab, and ±1 for dabrafenib and nivolumab + relatlimab to maximize treatment comparability and reduce individual patient outliers and label imbalance associated with our limited patient dataset.

Leiden cluster cell response labeling

For each Leiden cluster, the number of cells labeled resistant and sensitive was determined by examining the clinical and cell viability data. Cluster population proportions were then calculated relative to the total number of cell spectra assigned to the cluster, where the sum of resistant + sensitive + unlabeled cell spectra proportions = 1.

Feature importance

Raman wavenumbers importance were determined by analyzing the classification accuracy dropoff from spectra masking across all wavenumbers[12]. First, a 10-split stratified shuffle cross-validation (sklearn) was utilized with an 80:20 training:test split. Utilizing the random forest model discussed prior, we then determine the general classification performance as each wavenumber feature is perturbed with a Voigt profile of alpha=5, gamma=2 of the pre-transformed data for each test split. The average classification accuracy and standard deviation was then calculated for each feature. The Raman bands with the biggest effect on classification were selected by a descending sort of the largest peaks (SciPy) associated with 1-classification accuracy.

Construction and implementation of resistance determination model

A random forest multiclass classifier (sklearn) was constructed for each drug of interest using hyperparameters max_depth = 20, max_features = "sqrt", n_estimators = 275. Hyperparameters were selected by grid searching on the nivolumab training dataset and utilized for remaining determination models. Additionally, class_weight="balanced" was used to account for imbalanced training datasets. Commercial human cell lines and patient samples without additional treatments were used for our training and test datasets. We augmented the inhibitor training set with commercial cell line spectra. Each training patient sample was required to have 500+ spectra. Labels 0 (sensitive) and 1 (resistant) were annotated based on clinical observations and in vitro cell viability assessments. Cell viability cutoffs were set at 70% (targeted inhibitors) and 60% (immunotherapy) with edge cases (±2%) annotated sensitive. In summation, the first passages of patient samples PAT-52, PAT-54, PAT-63, PAT-73, PAT-93 and cell lines A375, SK-MEL-24, and SK-MEL-30 were utilized in our training set. Class probabilities from 0 to 1 as defined by class probabilities in our RF model were determined for each spectra, where values closer to 0 and 1 corresponded with more sensitive and more resistant, respectively. A binary label of resistant or sensitive could then be assigned based on a desired determination cutoff threshold for each drug protocol of interest. In our standard model, we chose 0.5 as our determination cutoff for the aggregated patient response. Labeling assignments for each treatment model were also calculated for determination cutoff ranges from 0 to 1 at 0.1 intervals to select optimized cutoffs. For each patient, 25,000 mini patients were generated by grouping 100 cell spectra randomly selected using uniform weighting from the patient superset. Drug resistance probability was predicted for each mini patient spectra using our pre-trained RFs, and the quantitative response of a mini patient was calculated by the mean of the spectra likelihoods. The patient response determination was then assessed by examining the median, 5th, and 95th percentile.

scRNA-seq analysis

Data were aligned with cellranger 9.0.0 to reference genome GRCh38-2024-A[13]. Data were imported into python as anndata objects for further analysis using the Scanpy[8,14]. Preprocessing was performed on each sample individually. Cells with fewer than 100 unique genes or more than 10% mitochondrial counts were removed. Doublets were removed using the DoubletDetection package with Louvain clustering, 20 iterations, 1e-16 p-value threshold, and 0.5 voter threshold[15]. Integration was performed with scVI using the top 2000 highly variable genes[16–18]. The model was trained for 100 epochs. Leiden clustering was performed at resolution 0.9. Differential expression

analysis was performed using the scVI differential expression module, and cells were manually annotated as tumor cells and fibroblast-like cells using canonical marker genes. One cluster that had a mixed gene expression profile was marked as unknown. The scANVI framework was then used for further integration using the scVI annotations as initial labels[19]. Leiden clustering was performed at resolution 1.0. Differential expression analysis was performed using the scVI differential expression module, and cells were manually annotated as tumor cells and fibroblast-like cells using canonical marker genes.

Statistical Analysis on Raman spectroscopy

Statistical analysis was performed at individual wavenumbers of interest, selected based on prior literature and preliminary spectroscopic evaluation. For each center wavenumber, intensities were integrated over ± 5 cm^{-1} window to account for spectral variability. Two complementary statistical approaches were employed to account for the hierarchical structure of the data (cells nested within biological replicates) and to provide robust assessment of treatment effects. Single-cell measurements (μ=243 cells, n=3 biological replicates per treatment) were analyzed using 1) one-way analysis of variance (ANOVA) with Tukey's HSD (scipy, statsmodels)[20] on biological replicate-averaged integrated intensities, and 2) linear mixed-effect models (LMMs) on single-cell data with biological replicate as random effect. LMMs were fitted using the Powell optimization method to ensure convergence. LMM p-values were adjusted for multiple testing using the Benjamini-Hochberg false discovery rate (FDR) procedure across all tests ($\alpha = 0.05$). Random effects variance was minimal, indicating high between-replicate consistency. Both approaches are reported for transparency; statistical significance defined as $p<0.05$.

Statistical analysis on intracellular lipid, protein, and phosphorylation quantification

Lipid: Comparison of multiple groups was performed by using one-way analysis of variance (ANOVA) with Dunnett correction. Protein: Sample protein concentrations were interpolated from a standard curve using GraphPad Prism (v. 10.6.0). Comparison of multiple groups was performed by using one-way ANOVA with Dunnett correction. Phosphorylation: Signal intensity was quantified using ImageJ 1.54 and plotted in GraphPad Prism (v. 10.6.0). Comparison of multiple groups was performed by using one-way analysis of variance (ANOVA) with Dunnett correction.

Supplementary Figures

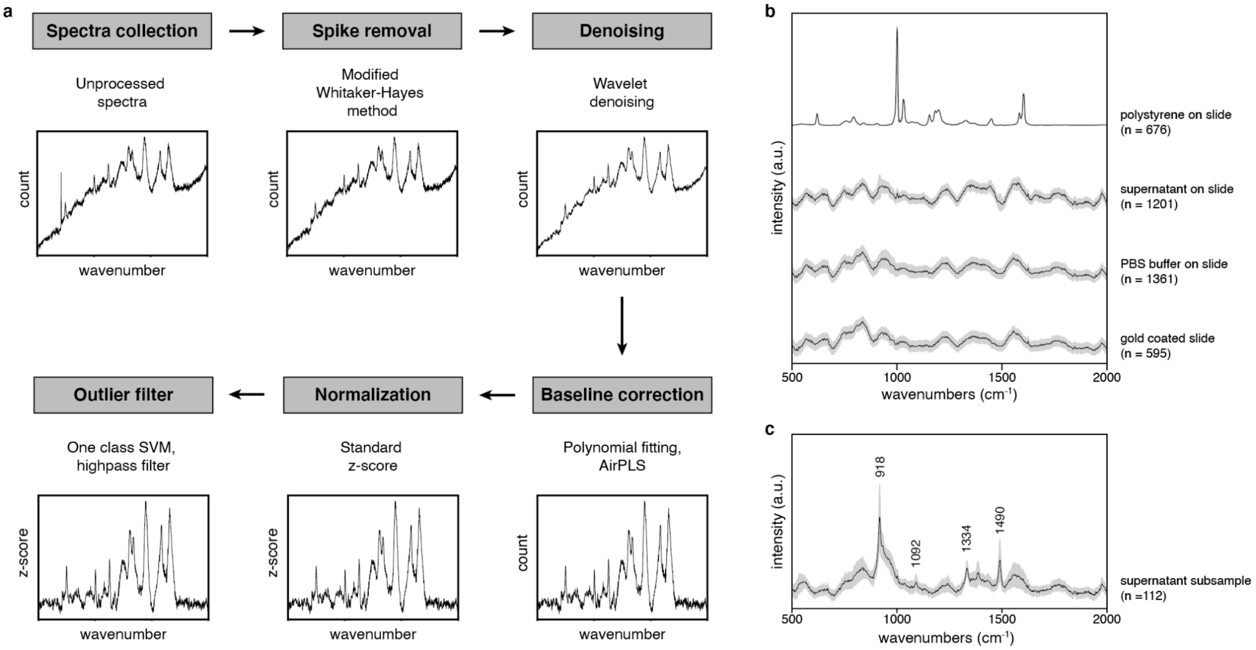

Supplementary Figure 1. Workflow of Raman spectra preprocessing. A) Representative workflow of the preprocessing steps taken for each spectrum. Preprocessing was essential to normalize and remove artifacts for cell spectra comparisons. An example spectrum of RAW264.7 is shown, highlighting the post-processed spectra after each step. B) Averaged spectra with ±1 standard deviation of post-processed background and outlier spectra acquired for our automated data filter step. The data filter step utilizes a combination of single class support vector machines (SVMs) and high-pass z-score values, and occurs after the preprocessing workflow highlighted in (A). C) A subset of spectra in the supernatant spectral set in (B) where major peak assignments are shared with polymers such as polyoxymethylene copolymer (POM-C) found in literature.

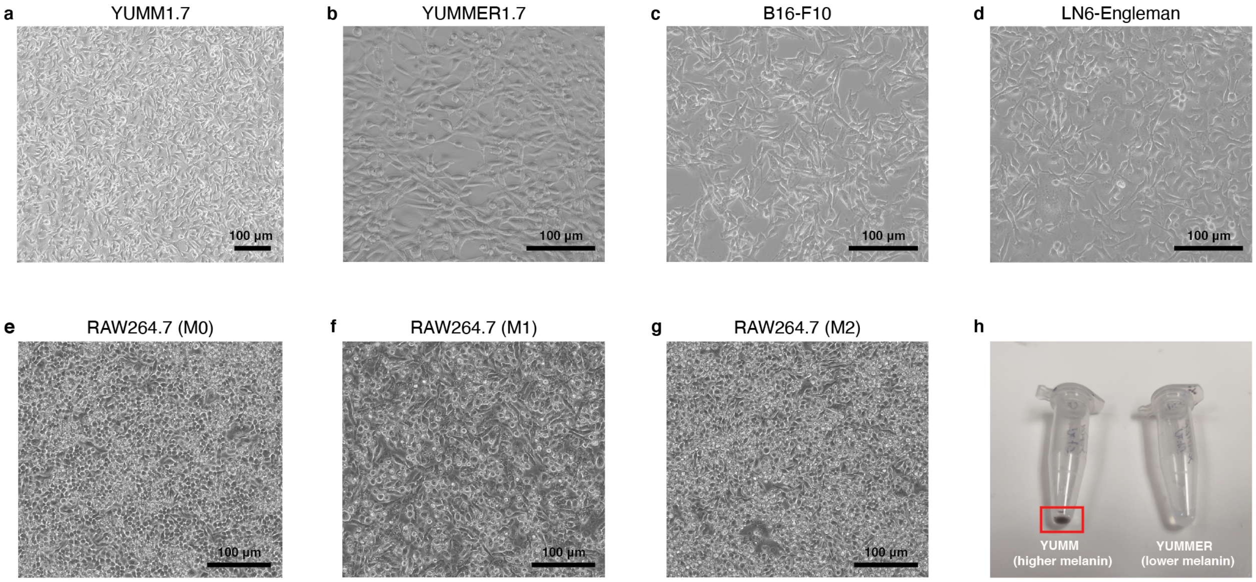

Supplementary Figure 2. Bright field images of mouse cells. A-G) Brightfield images of live murine cell lines. Images highlight morphological differences associated across cell types in cancer and immune cells and functional phenotypes in polarization states. Scale bar, 100 µm. H) Image of YUMM1.7 and YUMMER1.7 cell lines highlighting contrast in melanin pigmentation (higher melanin in the left tube highlighted with a red box), which was captured in our Raman analysis.

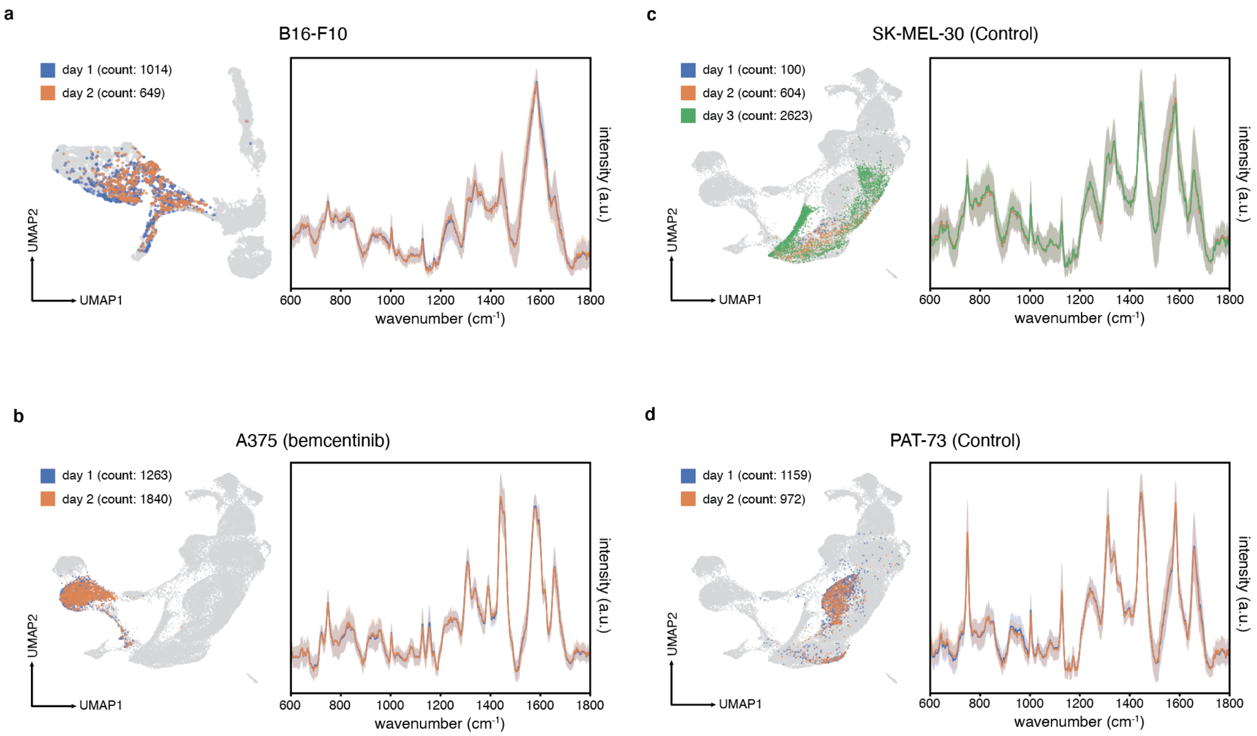

Supplementary Figure 3. Day to day variations in Raman spectra acquisition. Select mouse cell lines and human patient-drug treatment samples were measured across multiple days to examine spectra variability relating to sample preservation, environmental fluctuations, and instrumentation inconsistencies for (a) B16-F10, (b) A375-bemcentinib, (c) SK-MEL-30-control, and (d) PAT-73-control. Findings show consistent overlays across our embedded space (left) for a given sample-treatment protocol, and average spectra well within the ±1 standard deviation (right).

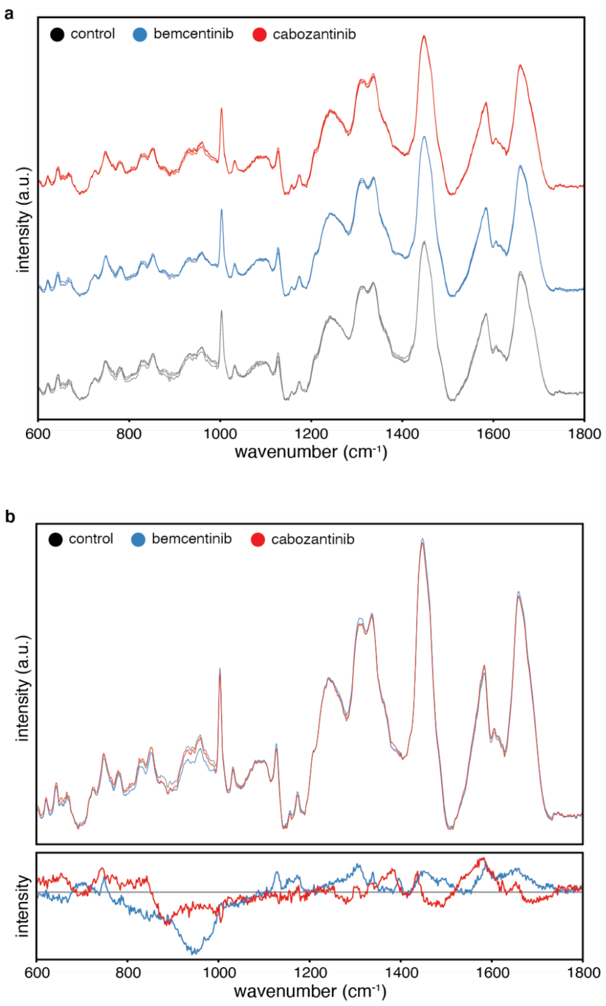

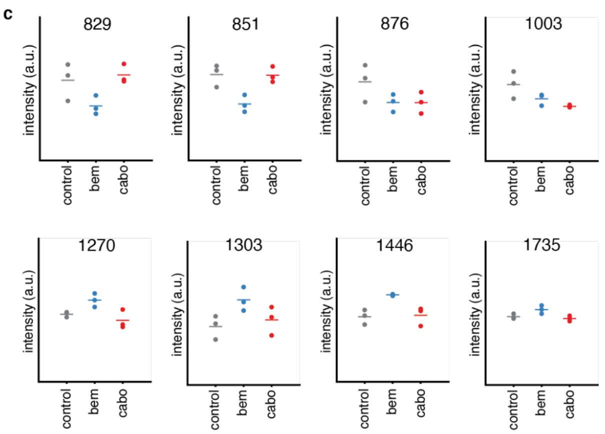

d

wavenumber (cm-1)	1-way ANOVA	Post-hoc		Linear Mixed-Effects (FDR-adj)	
		Tukey (bem)	Tukey (cabo)	LMM-FDR (bem)	LMM-FDR (cabo)
829	0.060	0.122	0.878	0.055	0.673
851	**0.013**	**0.019**	0.996	**0.004**	0.993
876	0.173	0.222	0.220	0.090	0.090
1003	**0.053**	0.185	**0.048**	0.071	**0.010**
1270	**0.029**	0.103	0.550	**0.029**	0.359
1303	0.096	0.095	0.808	**0.029**	0.671
1446	**0.014**	**0.019**	0.966	**0.005**	0.869
1735	**0.031**	0.078	0.762	**0.029**	0.600

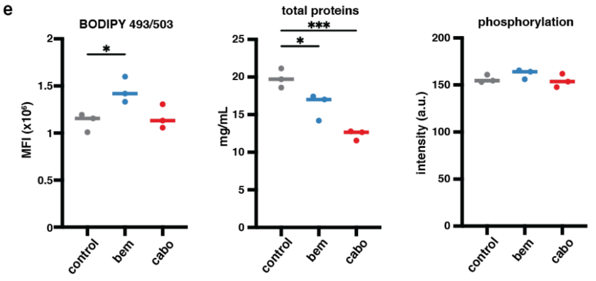

Supplementary Figure 4. Lipid and protein quantification of post-treatment YUMMER1.7 cells. A) Mean Raman spectra (μ=243 cells) of control (black), bemcentinib-treated (blue), and cabozantinib-treated (red) YUMMER1.7 cells (n=3 biological replicates) in close agreement within treatment groups. B) Top: Mean Raman spectra of cell response to treatment groups (μ=729 cells). Bottom: Spectral differences of post-treated samples relative to untreated (control). C) Intensity distributions at representative wavenumbers showing post-treatment differences. Lipid-only (1735 cm^{-1}) and lipid-dominant (1270, 1303, 1446 cm^{-1}) peaks commonly used in composition analysis showing post-bemcentinib spectra with increased intensities and post-cabozantinib spectra with relatively same intensity to control[21–26]. Protein only peaks (1003, 876 cm^{-1}) showing intensity decreases across post-bemcentinib and post-cabozantinib spectra, with a larger decrease on average for post-cabozantinib[22,24,27,28]. Tyrosine-related peaks known as fermi resonance bands (829, 851 cm^{-1}) showing an intensity decrease in post-bemcentinib, with no doublet collapse or change in tyrosine-associated 1205 cm^{-1} intensities likely indicating reduced phosphorylation-related changes[29–31]. D) Statistical analysis of select Raman spectral peaks. One-way ANOVA tested overall differences among protocols. Post-hoc pairwise comparisons performed using Tukey's HSD (correcting for three comparisons) and linear mixed-effect models (LMM) with false discovery rate (FDR) correction across all tests (16 tests, Benjamini-Hochberg procedure). Bold values indicate $p<0.05$. See Supplementary Table 4 for Raman peak assignments. E) Cell lipid and protein quantification. Left: Intracellular neutral lipid content by BODIPY 493/503 flow cytometry showing post-bemcentinib cells having elevated lipid content. Middle: Total protein quantification by BCA assay reveals decreased protein in both post-bemcentinib and

post-cabozantinb persistent cells. Right: Western blot analysis using a phospho-motif antibody showing no significant differences among treatment groups.

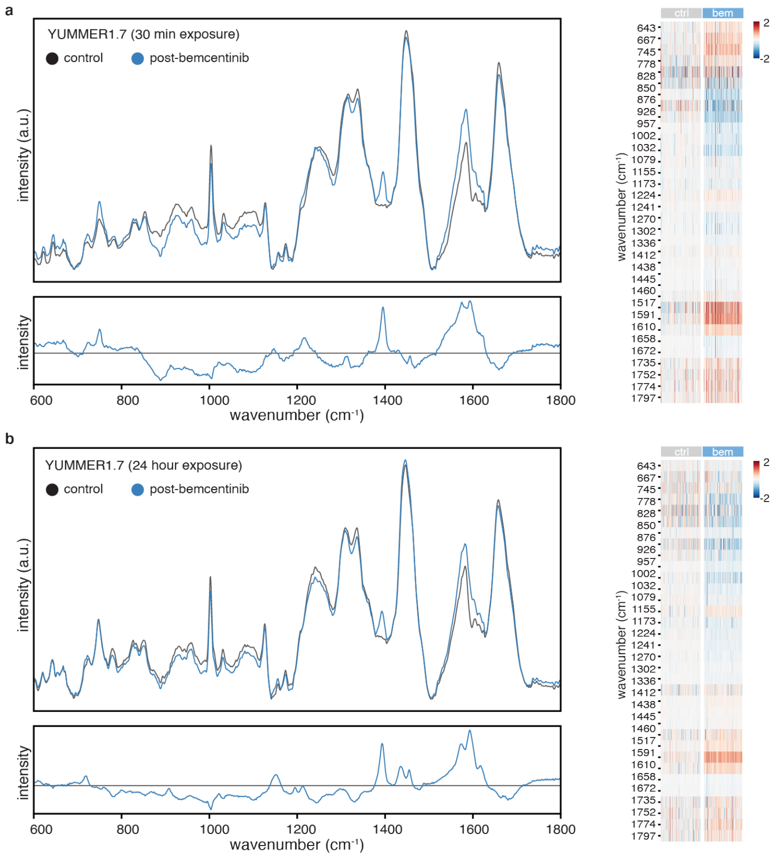

Supplementary Figure 5. Temporal dynamics of Raman spectral changes in bemcentinib-treated YUMMER1.7 cells. YUMMER1.7 cells were exposed to bemcentinib for 30 minutes (a) or 24 hours (b). Left panels: Mean normalized Raman spectra of control (black) and post-bemcentinib (blue) cells (top) with corresponding difference spectra (bottom, post-treatment minus control). Right panels: Differential Raman intensities across select wavenumbers, with cells grouped by treatment conditions. Color scale represents fold change from -2 to +2. Pronounced spectral changes were observed at 30 minutes followed by partial recovery towards baseline at 24 hours across lipid (958, 1447, 1658 cm^{-1}), protein (750, 930, 958, 1003, 1030, 1241, 1447, 1658 cm^{-1}), and nucleic acid (750, 1241, 1337 cm^{-1}) peaks. This temporal trajectory is consistent with initial acute cellular stress response followed by adaptive remodeling.[32–36] Temporal trajectories at nucleic acid-associated bands (780-800 cm^{-1}) differed from the intensity changes characteristic of apoptotic cells, supporting an adaptive remodeling interpretation.[37–40] Spectral peaks reflective of remnant bemcentinib which was seen in prior analysis (Supplementary Figure 6) demonstrate Raman's high sensitivity.

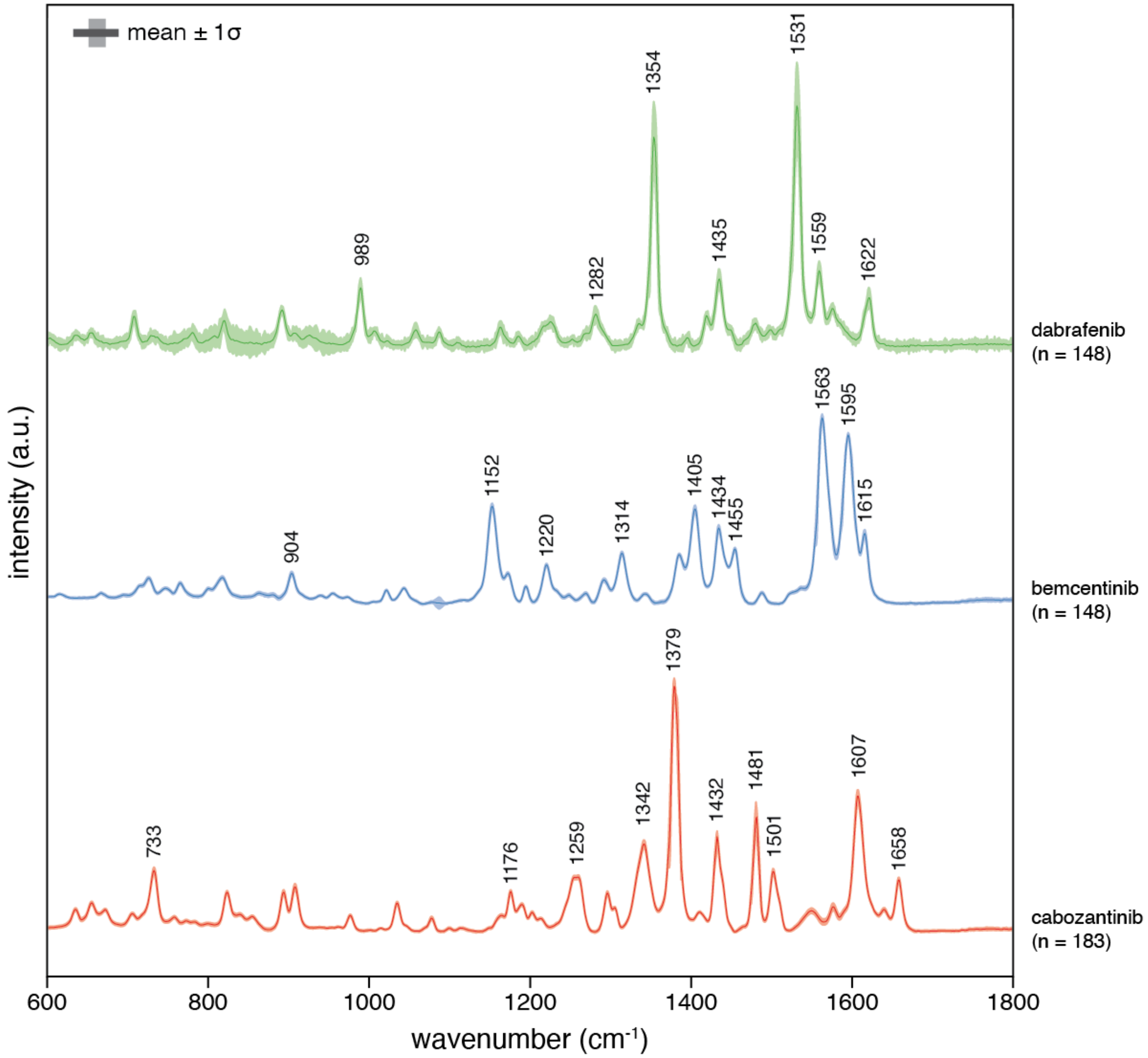

Supplementary Figure 6. Raman spectra of molecular inhibitors. Mean normalized spectra and ±1 standard deviation for targeted inhibitors cabozantinib, bemcentinib, and dabrafenib. Wavenumber assignments are annotated for each averaged spectra.

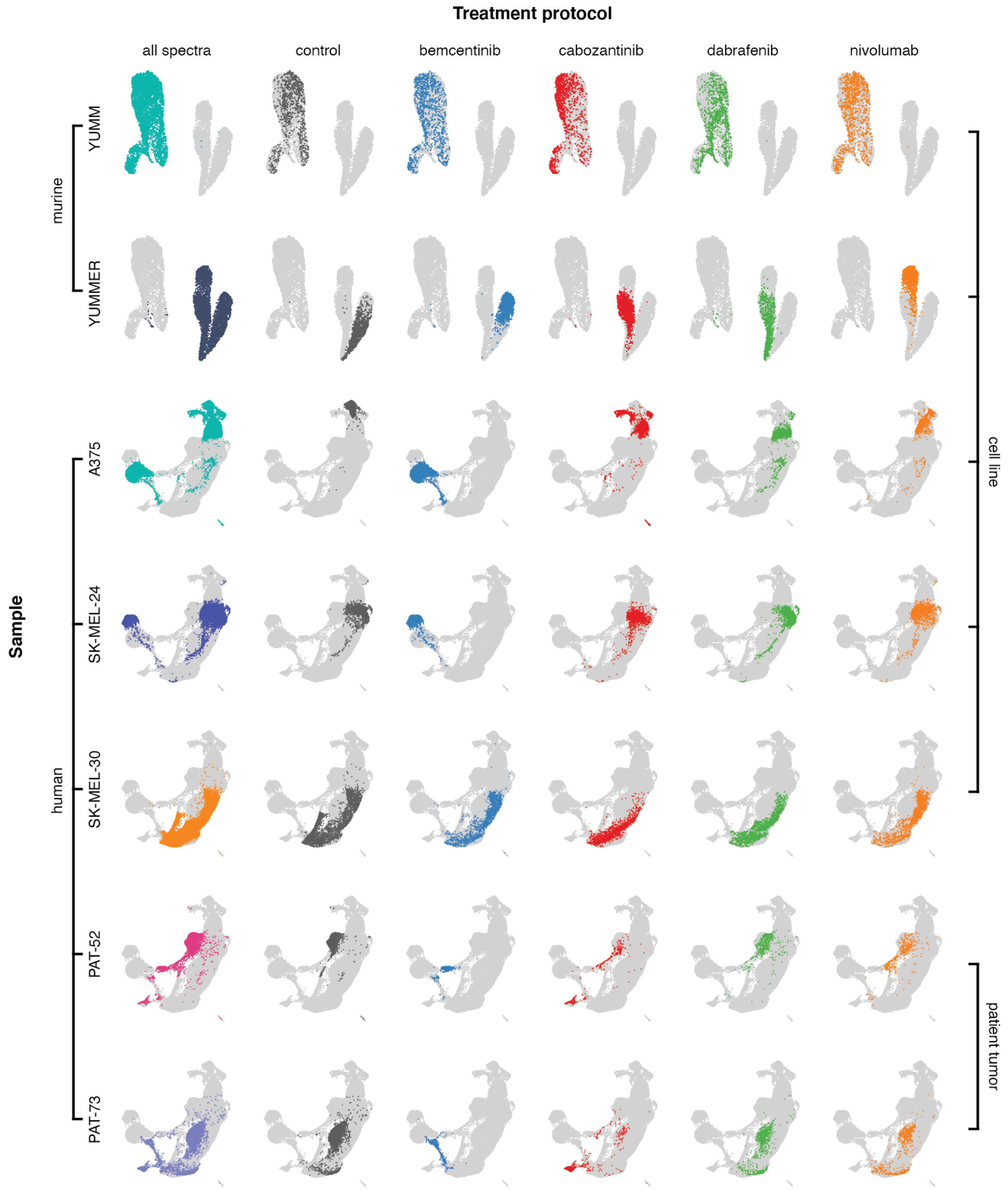

Supplementary Figure 7. One-vs-all cell spectra for mouse and human samples. Latent space localizations of mouse and human line samples discussed in Figure 3 and 4. For each sample, spectra are highlighted with respect to cell procurement source (row) and treatment protocol (column). The first column encompasses all affiliated cell spectra acquired for a given sample, and each successive column represents either an untreated cell set (control) or persistent cells measured following a treatment protocol.

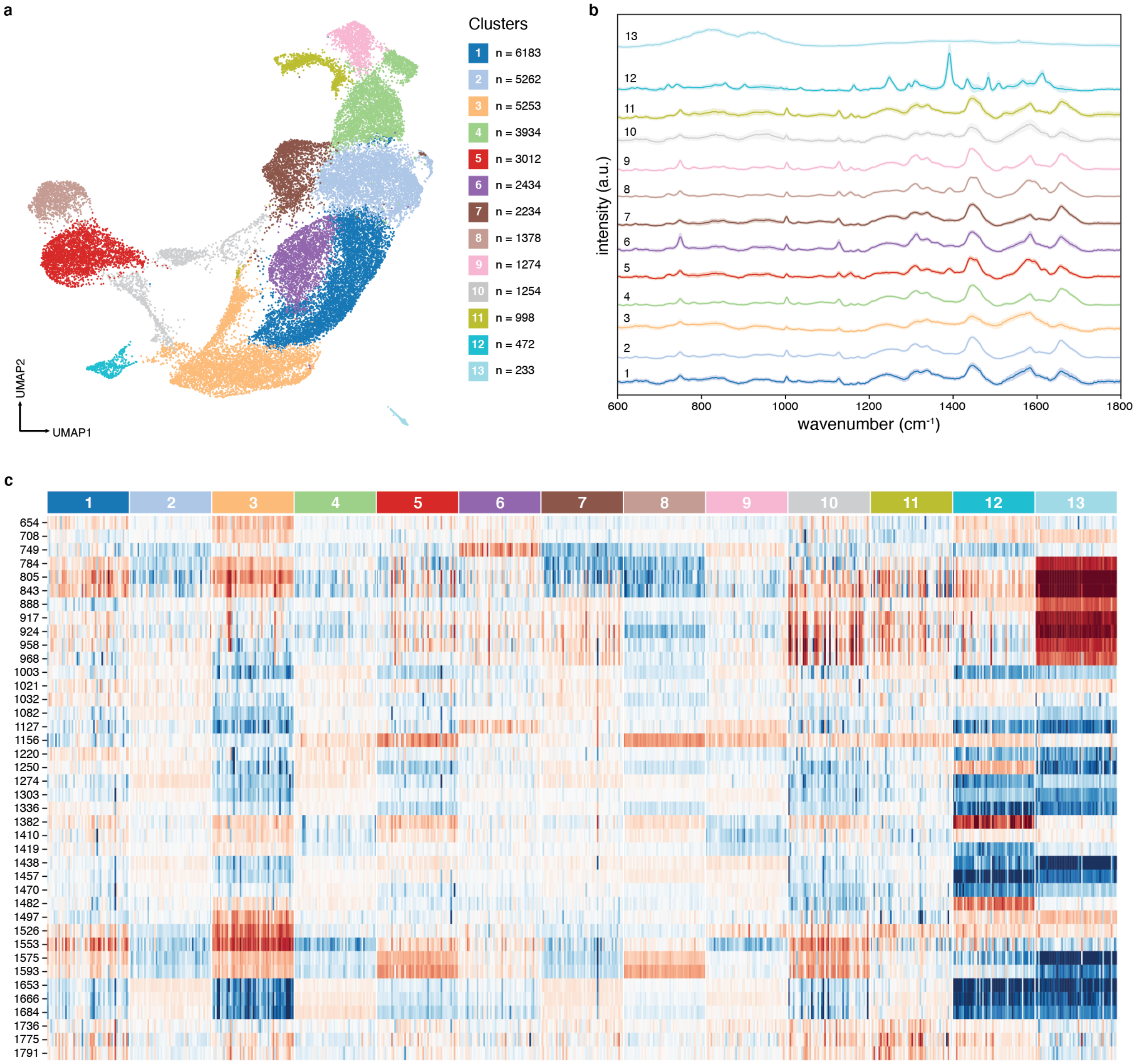

Supplementary Figure 8. Leiden clustering of human samples. A) UMAP embedding of Raman profiles from human melanoma response dataset grouped by Leiden clustering. Hyperparameters selected were nearest neighbors = 25, minimum distance = 0.3, number of epochs = 500, and Leiden resolution = 0.3. B) Mean normalized spectra and ±1 standard deviation across each Leiden grouping. Note, cluster 13 shows typical spectra from our substrate, while cluster 12 shows major bands found in spectra observed in cabozantinib. C) Differential wavenumber intensities of the median fold change across Leiden clusters showing distinct Raman "expression" for each cluster.

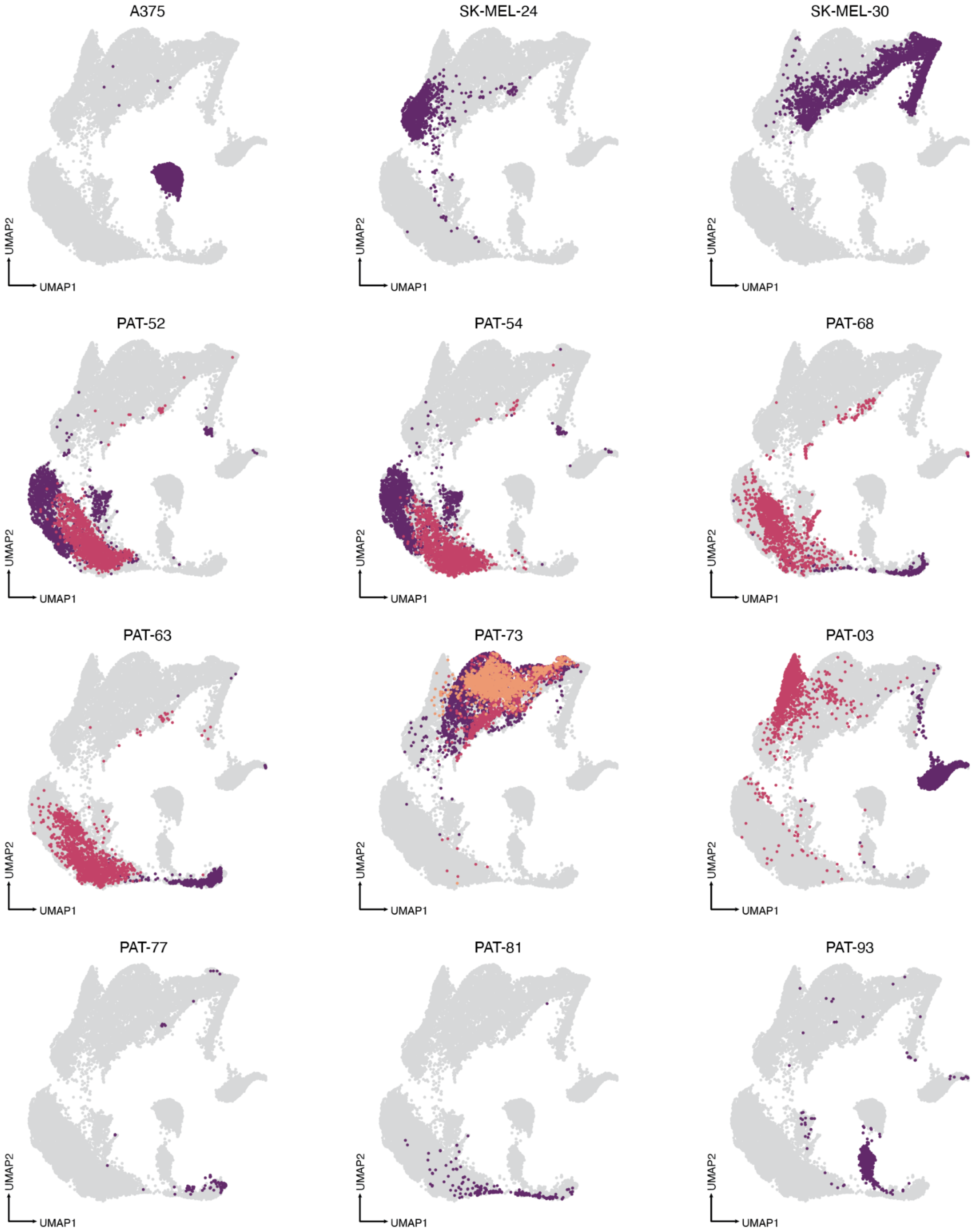

Supplementary Figure 9. Passage to passage variations for select patient samples. UMAP embedding of Raman profiles from patient melanoma and commercial cell lines. First passage is colored purple, second passage colored red, and third passage, if applicable, is colored orange. Two passages were analyzed for all patient samples above,

except for PAT-73 in which three passages were analyzed. Commercial cell lines are represented in the first row, primary tumors in the second, and immunotherapy-refractive patients in the remaining rows.

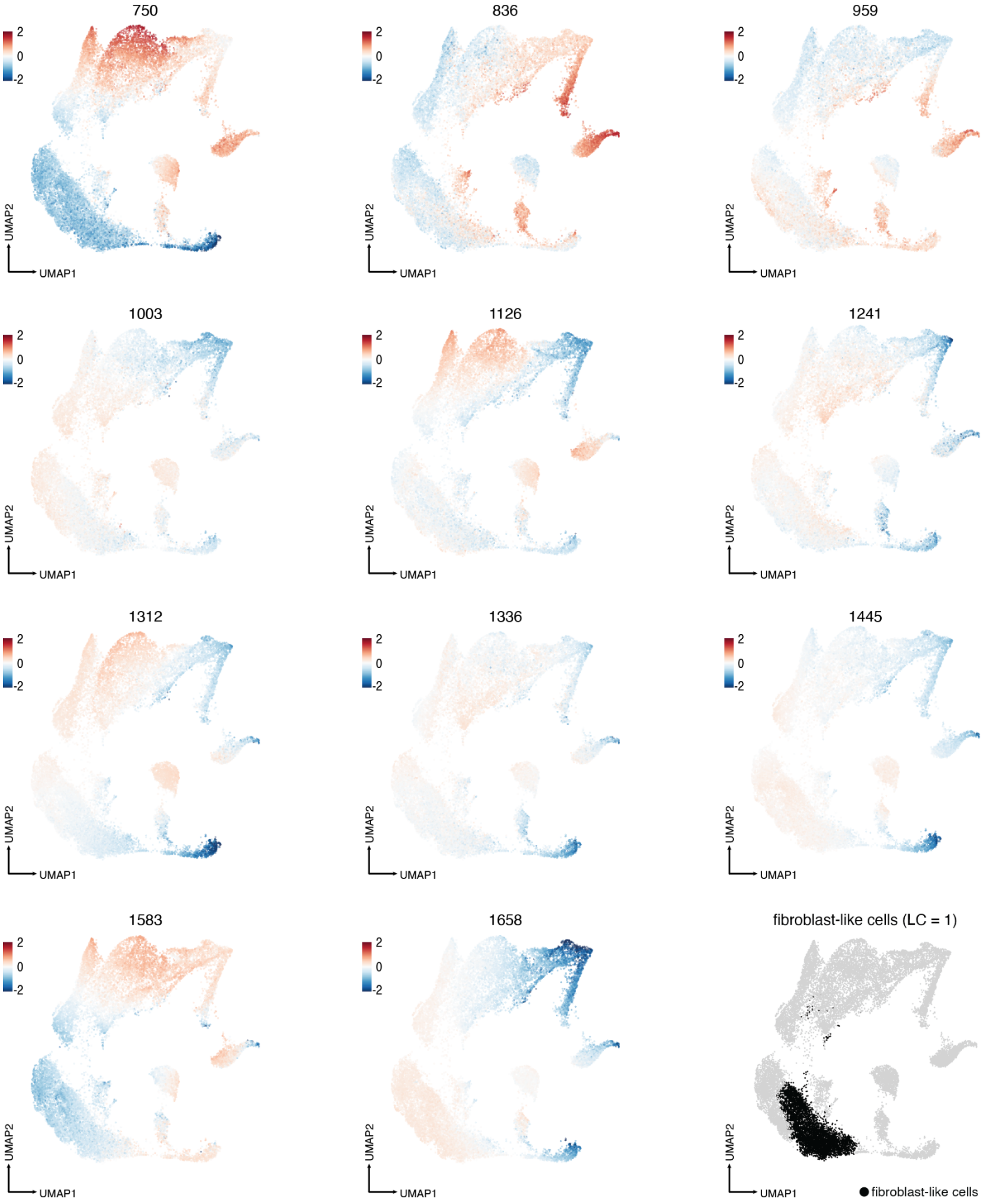

Supplementary Figure 10. Raman intensity fold change levels for patient samples by UMAP. Median fold change across select highly variant wavenumbers for our patient dataset, showing elevated or reduced “expression” across localized regions in latent space. Fibroblast-like cell profiles (cell spectra highlighted in black in the last subplot and corresponded to Leiden grouping 1) had elevated “expressions” across 1658 and reduced “expressions” across 750, 1312, and 1583 cm^{-1}.

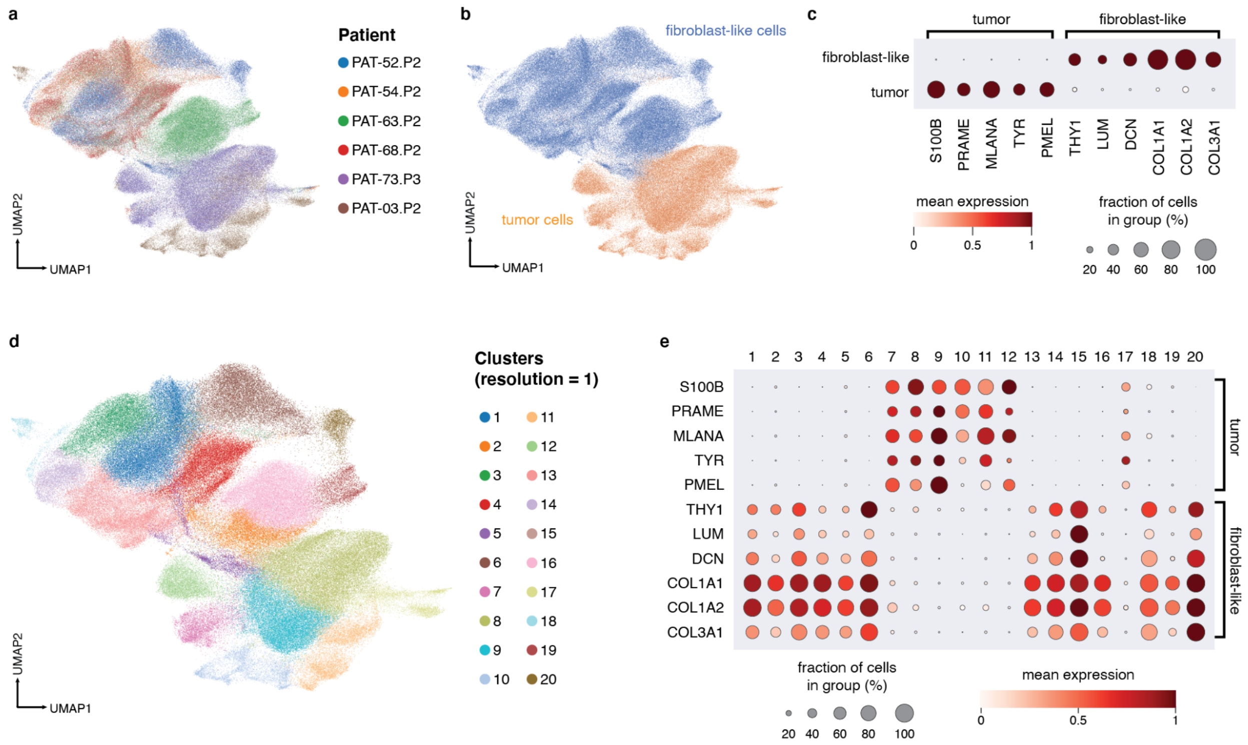

Supplementary Figure 11. Single-cell RNA sequencing of patient melanoma samples. Latest passages from samples PAT-52, PAT-54, PAT-63, PAT-68, PAT-73, and PAT-03 were sequenced. A) Transcriptomic UMAP colored by patient. B) UMAP colored by cell type based on canonical marker genes. C) Dotplot of marker genes associated with tumor-like and fibroblast-like features. D) Leiden clustering at resolution 1.0. E) Dotplot of marker genes grouped by Leiden cluster. We observe bimodal distribution in the expression of these markers.

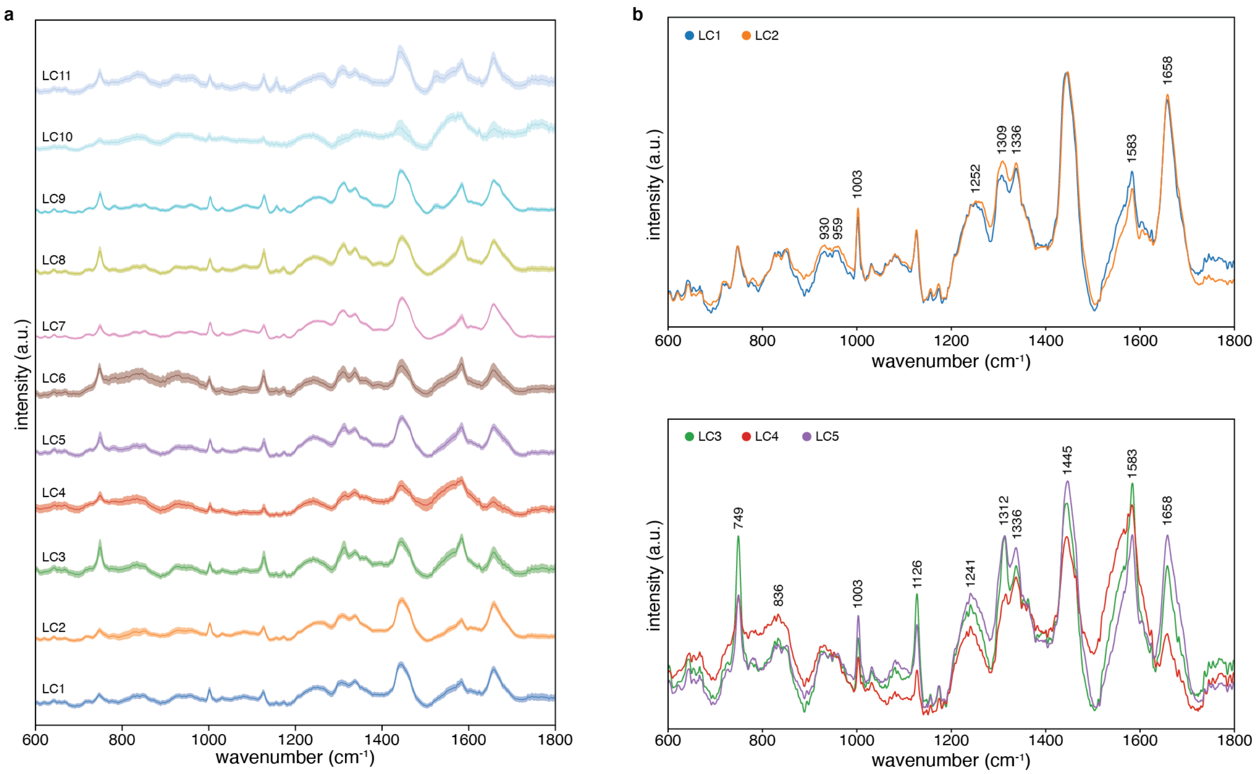

Supplementary Figure 12. Leiden clustering for human cell lines and patient samples. A) Mean normalized Raman spectra and corresponding ±1 standard deviation of cells residing in each Leiden cluster (resolution = 0.5) from Figure 5a. B) Spectra comparison between cluster 1 and 2, highlighting differences between fibroblast-like cells (LC1) and tumor cells (LC2). C) Spectra comparison between clusters 3-5, highlighting large phenotype heterogeneity across tumor cells.

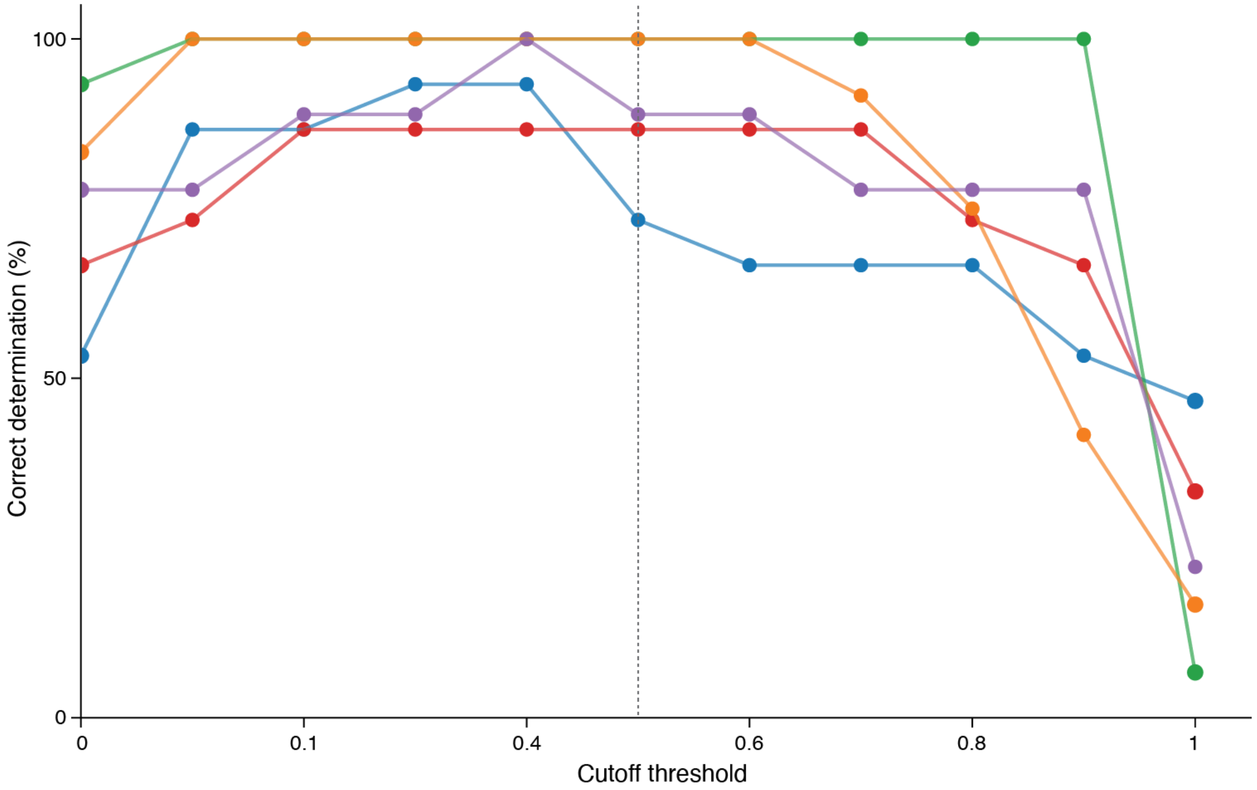

Supplementary Figure 13. Response determination accuracy vs. threshold selection. Examination of response determination accuracy of our prediction models across our patient samples as we vary the cutoff threshold (designating resistant and sensitive labels) of the median mini patient response. Assessment accuracy benchmarked against clinical outcomes and *in vitro* cell viability was determined for each drug classifier at 0.1 cutoff intervals. A 0.5 cutoff (dotted, grey line) was selected for all drugs of interest in our main analysis. For cabozantinib (red), dabrafenib (green), and nivolumab (orange) classifiers, the chosen cutoff threshold of 0.5 for our analysis provided the highest assessment accuracy. For bemcentinib (blue) and nivolumab + relatlimab (purple) classifiers, better assessment accuracies were found at lower cutoff values.

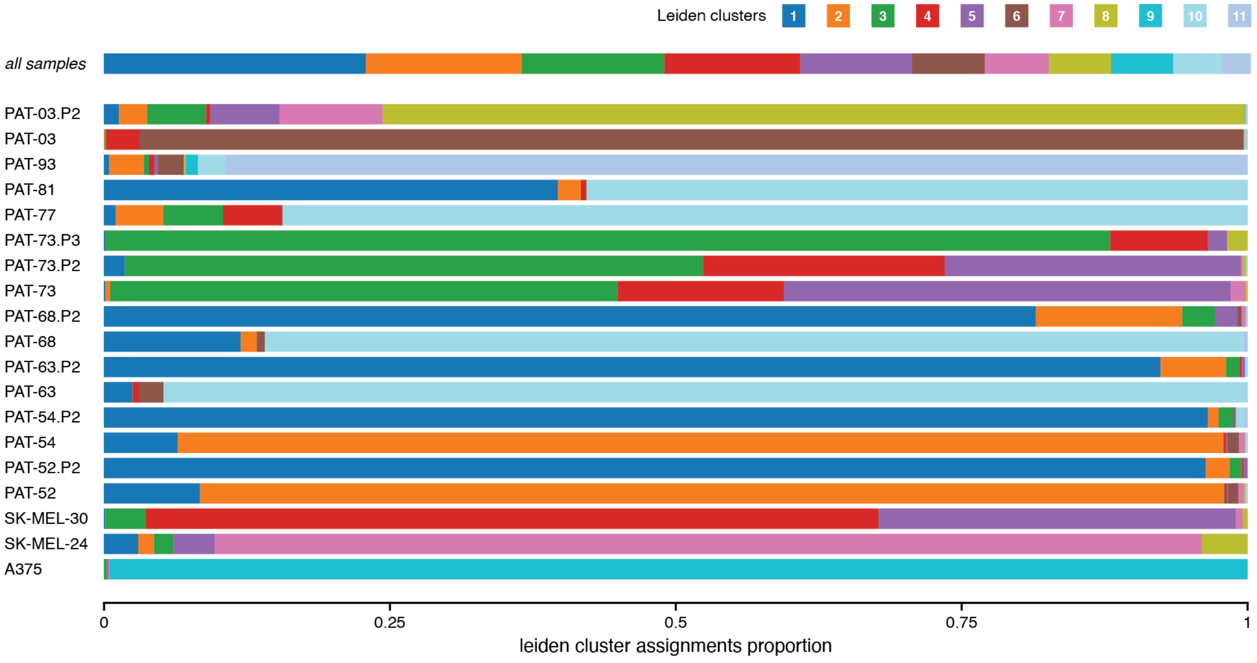

Supplementary Figure 14. Leiden cluster designation for patient-derived samples. Leiden cluster assignment proportions across each sample utilized in our patient prediction workflow, as well as the leiden cluster distribution encompassing our global dataset.

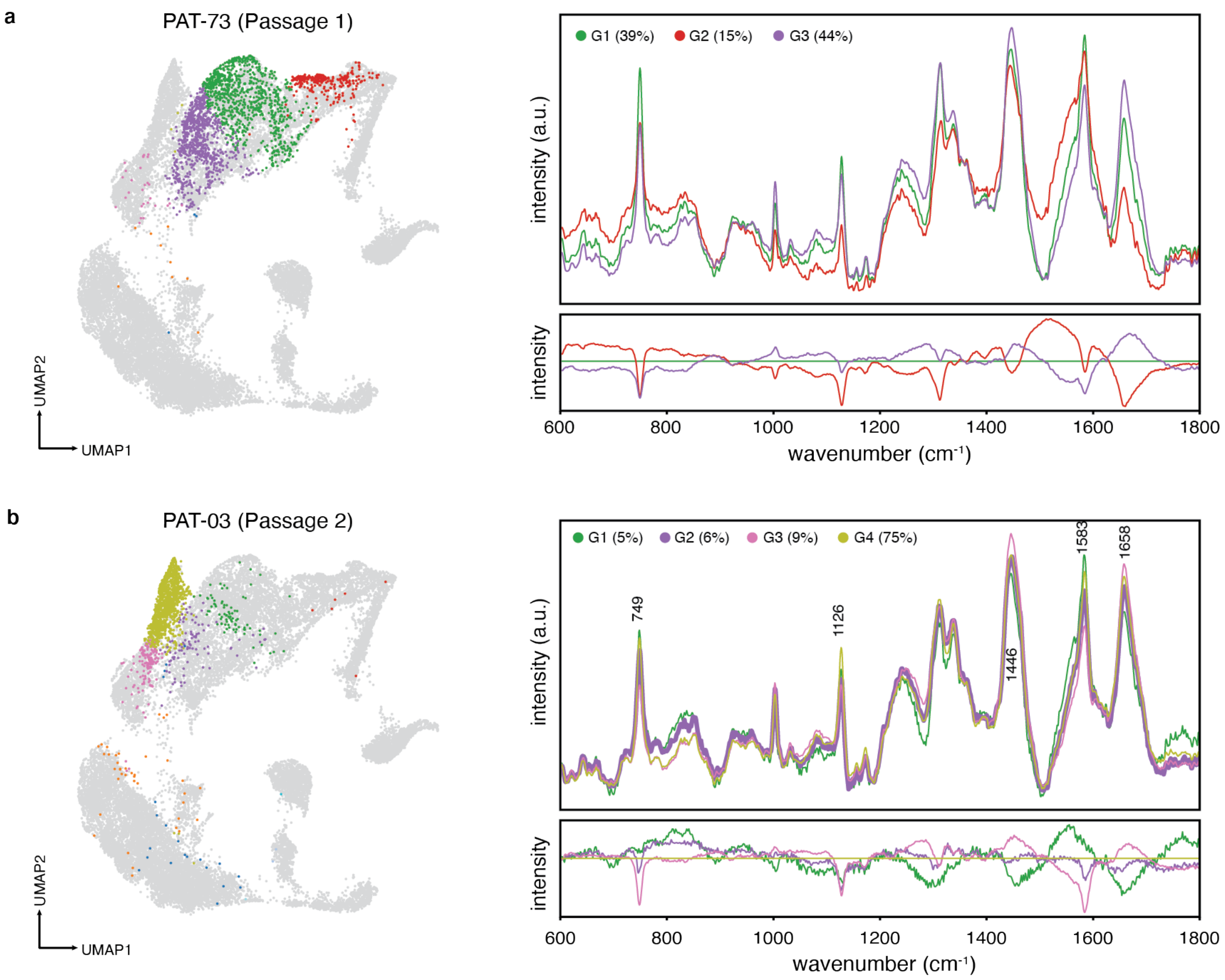

Supplementary Figure 15. Patient samples cell subpopulations. Select patient samples PAT-73 Passage 1 (A) and PAT-03 Passage 2 (B) showing intratumoral heterogeneity based on Leiden cluster assignments. For each section, Left: UMAP embedding of all measured cell spectra, colored by the assigned Leiden cluster found in Supplementary Figure 14. Right: Above is the mean average spectra of major groupings where a major group is defined as encompassing >5% of patient spectra, and bottom is the spectral differences among groups relative to largest grouping below. The % of patient spectra belonging to a major grouping is designated in the legend of each subfigure. Major differentiating peak assignments of (A) can be found in Supplementary Figure 12, and band assignments of 749, 1003, 1126, 1252, 1446, 1583, and 1658 cm^{-1} are identified for (B). Band assignments can be found in Supplementary Table 4.

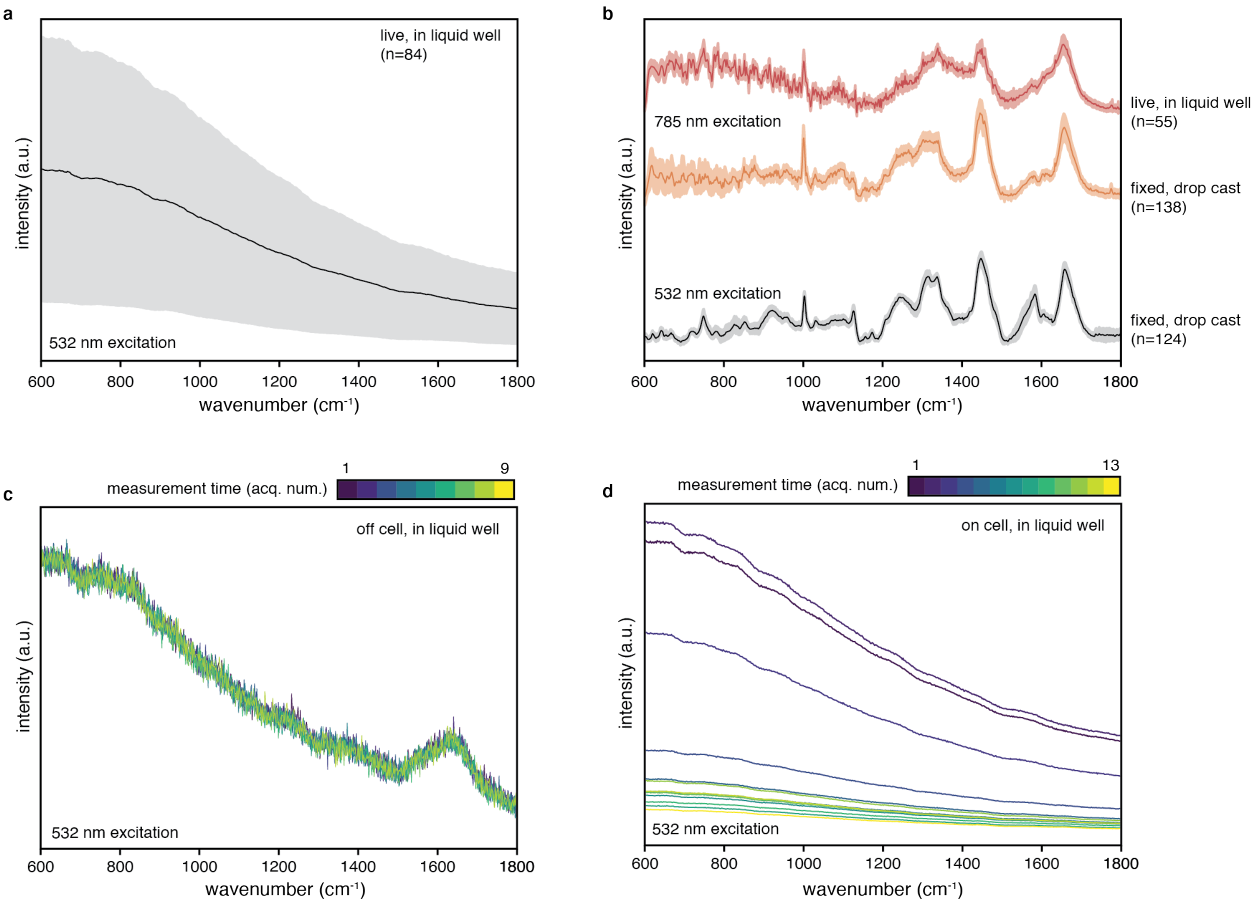

Supplementary Figure 16. Cell spectra differences across preparation conditions. YUMMER1.7 cells were prepared with different protocols for Raman measurements: 1) fixed and drop casted, and 2) live and in solution. A) Despiked and denoised average live cell spectra with ±1 standard deviation (n=84) under 532 nm excitation, showing high background fluorescence signal from the glass cover slip and autofluorescence of cells in PBS. Our fixed, drop-casted cell spectra exhibited weaker fluorescence signal. B) Averaged normalized spectra with ±1 standard deviation of live in suspension (n=55, red) and fixed, drop-casted (n=138, orange) cells under 785 nm excitation exhibited major spectral differences at 1000, 1250, 1312, 1341, 1447, 1580, and 1658 cm^{-1}, corresponding to lipid and protein bands likely arising from the presence of formaldehyde adducts, matching prior studies[41]. 785 nm excitation reduced fluorescence signal observed at shorter wavelengths. Averaged spectra with ±1 standard deviation of post-processed fixed and drop-casted cells (n=124, black) under 532 nm excitation serve as reference. Peak differences in our fixed spectra at 532 nm and 785 nm can be attributed to variations in vibration resonance enhancements that are excitation wavelength dependent. C, D) Time series (sequential) acquisitions of live in suspension samples at the same coordinate point, with each successive measurement mapped to an acquisition number. Off-cell spectra (C) showed minimal background signal, whereas on-cell spectra had high background that was reduced with successive measurements, likely from autofluorescence photobleaching[42].

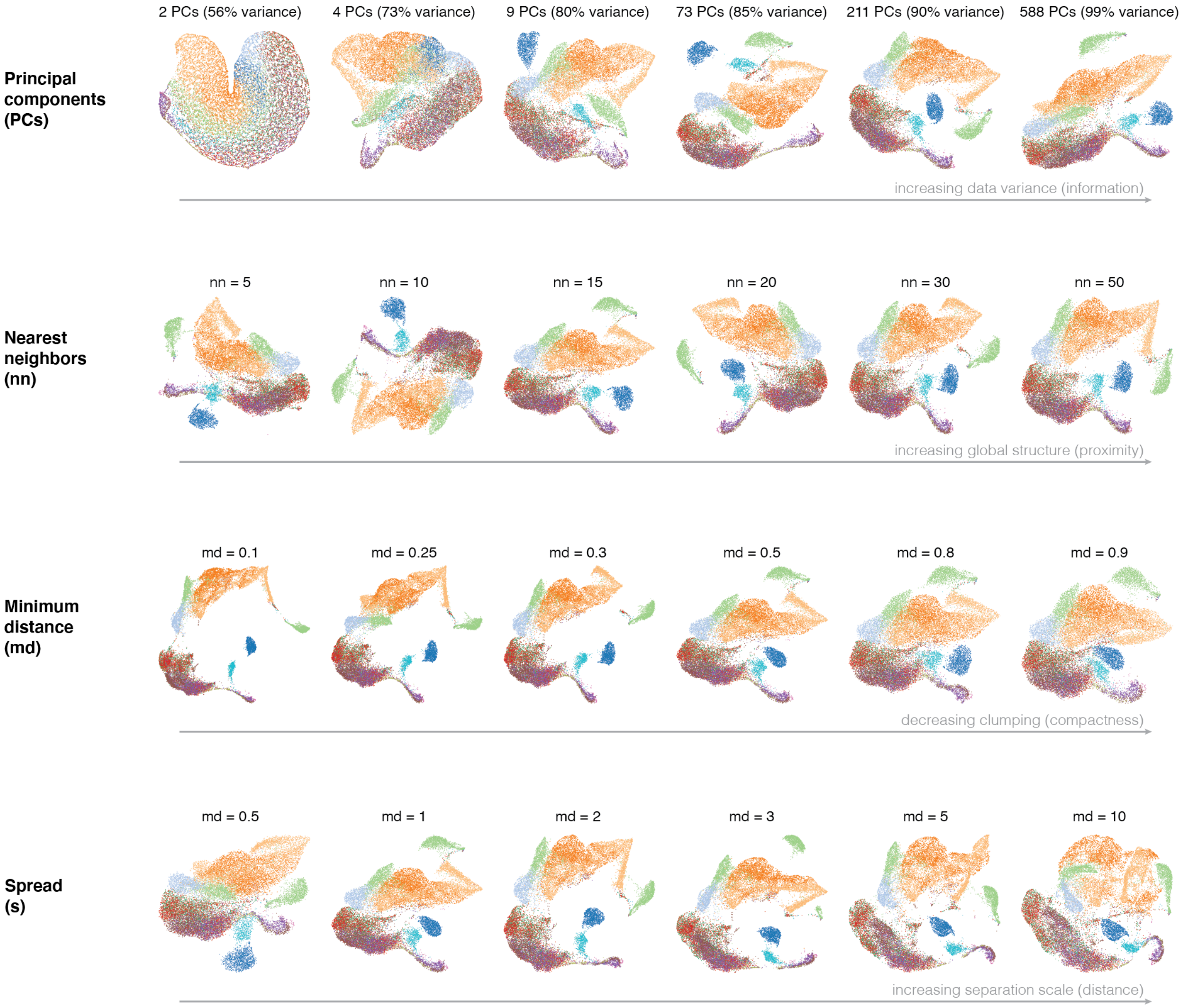

Supplementary Figure 17. Hyperparameter effects on our data clustering and analysis. Color corresponds to sample origin as defined in Figure 5 in the main text. Increasing the number of principal components retained more information and improved cell cluster separability. Increasing nearest neighbors examined more interconnectivity and preserved global structuring over local structures. Increasing minimum distance raised the lower bound of embedding space distance among points and decreased cell spectra crowding. Increasing spread enlarges the distribution of distances and affects the perceived distance between spectra.

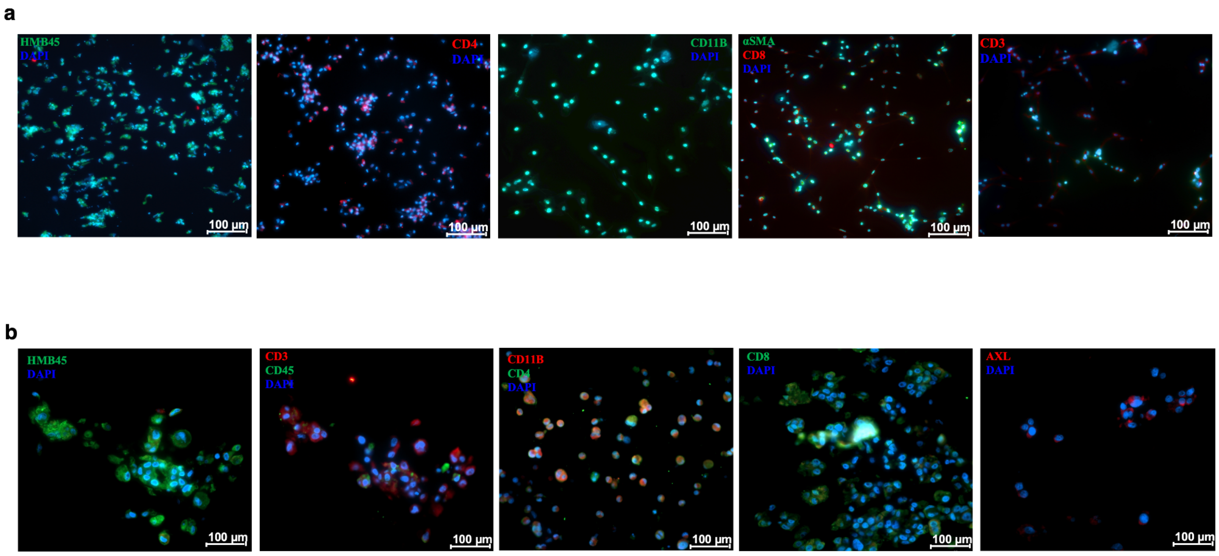

Supplemental Figure 18. Immunofluorescence of patient samples. Melanoma patient-derived samples retain immune cell composition and other cell types. A) PAT-52 patient-derived cells were allowed to grow for 7 days without passaging, and immunofluorescent tagging was applied with markers HMB-45, CD4, CD11B, α-SMA, CD8, and CD3. Scale bar, 100 µm. B) Images of PAT-73 patient-derived cells were allowed to grow for 7 days without passaging and immunofluorescent tagging was applied with markers HMB-45, CD3, CD45, CD11B, CD4, CD8, and AXL. Scale bar, 100 µm.

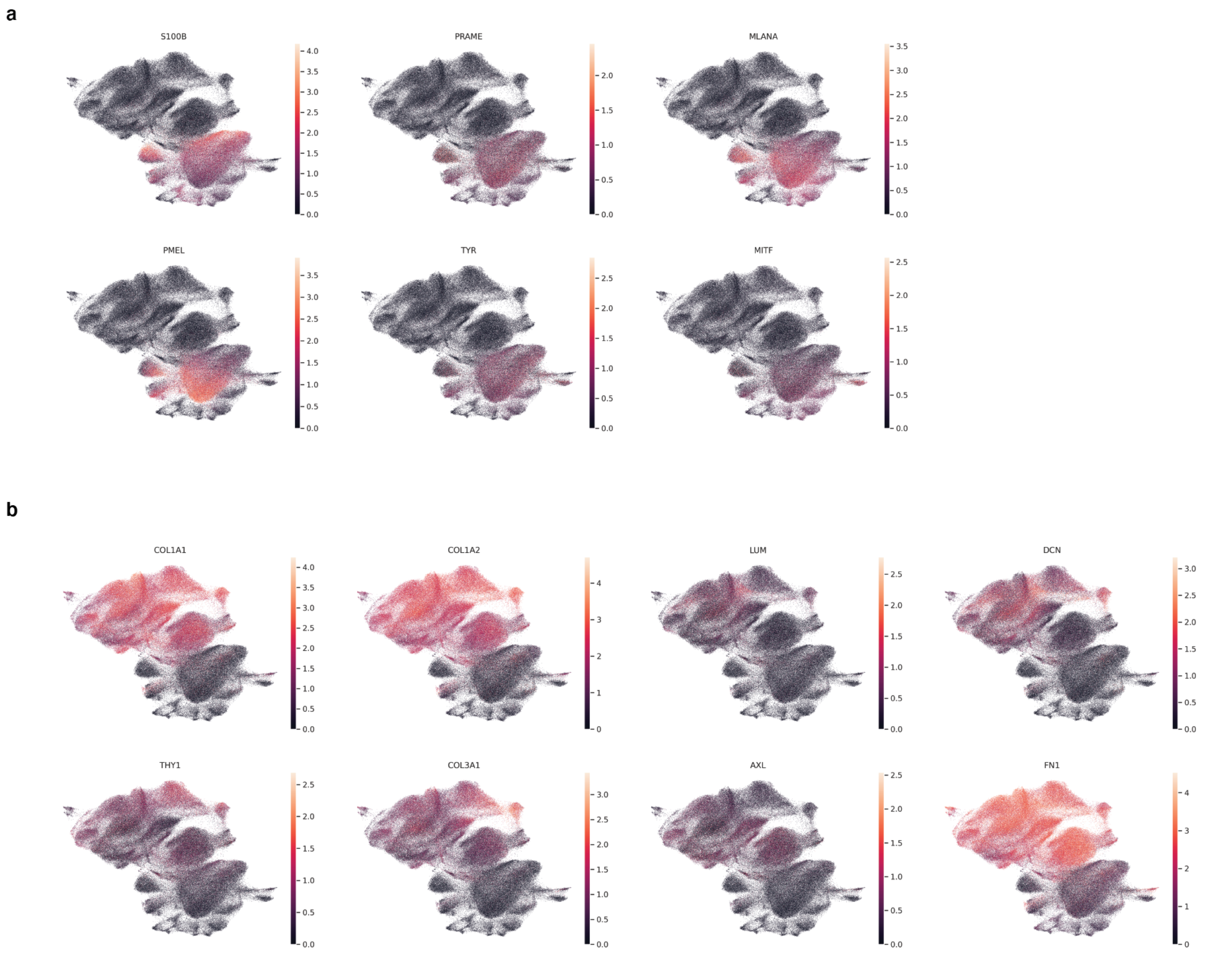

Supplementary Figure 19. Feature plots of patient melanoma samples. A) UMAP of our scRNA-seq dataset colored by marker genes S100B, PRAME, MLANA, PMEL, TYR, and MITF. The clusters with higher expressions of such genes are suggestive of tumor cells. B) UMAP of our scRNA-sequenced dataset colored by marker genes COL1A1, COL1A2, LUM, DCN, THY1, COL3A1, AXL, and PN1. The clusters with higher expressions of such genes are suggestive of fibroblast-like cells.

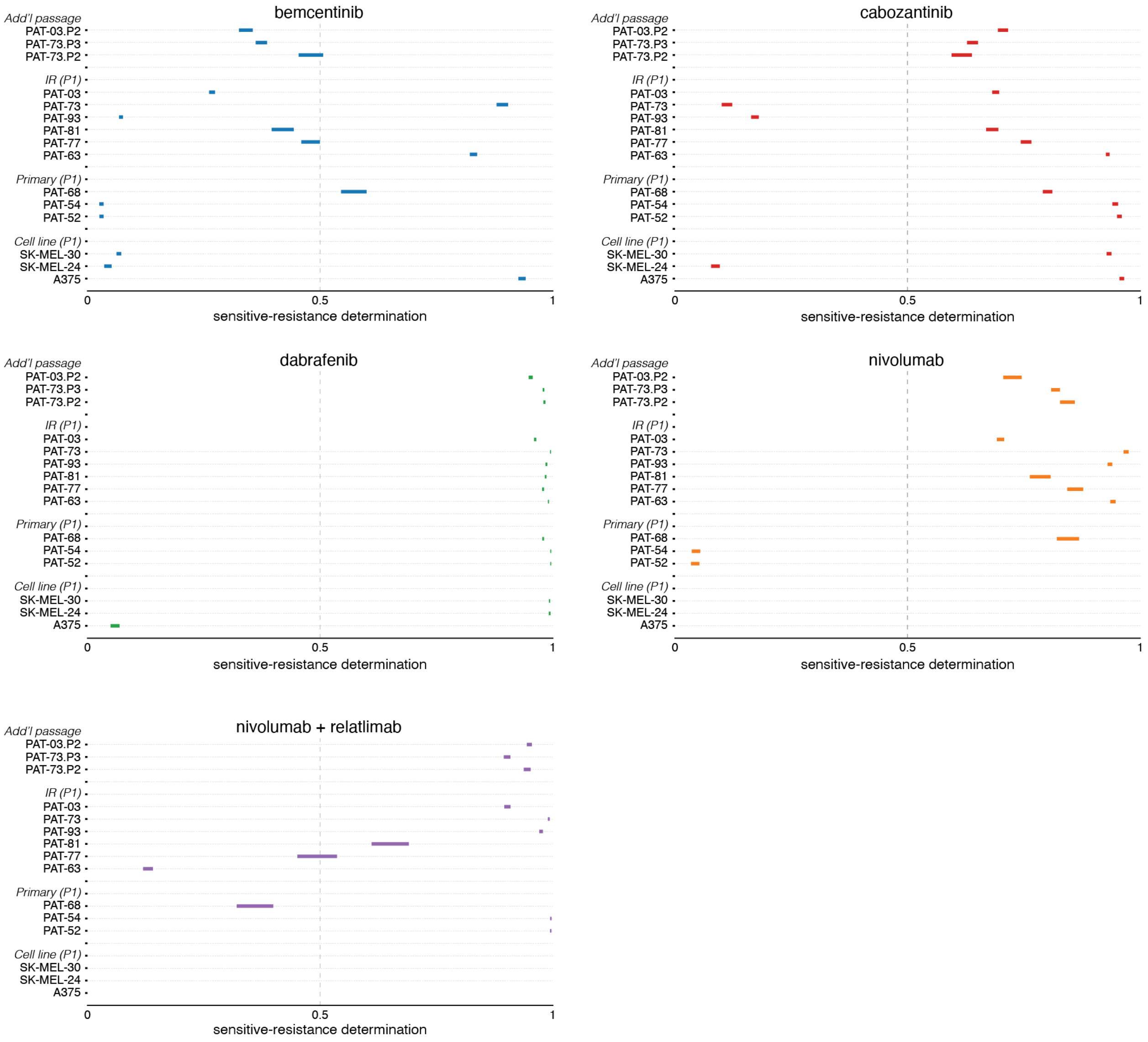

Supplementary Figure 20. Percentile distribution of mini patient responses. Response determination distribution for all mini patients sampled in each patient-derived cell line (seen and unseen by our ML model) across targeted and immunotherapy regimes. Bars represents the 5th to 95th percentile of the mini patient response.

References

1. Whitaker, D. A. & Hayes, K. A simple algorithm for despiking Raman spectra. *Chemometrics and Intelligent Laboratory Systems* **179**, 82–84 (2018).
2. van der Walt, S. *et al.* scikit-image: image processing in Python. *PeerJ* **2**, e453 (2014).
3. Zhang, Z.-M., Chen, S. & Liang, Y.-Z. Baseline correction using adaptive iteratively reweighted penalized least squares. *Analyst* **135**, 1138–1146 (2010).
4. Haque, A. *Feature Engineering & Selection for Explainable Models: A Second Course for Data Scientists*. (Lulu.com, 2022).
5. Negri, L. H. & Vestri, C. *Lucashn/peakutils: v1.1.0*. (Zenodo, 2017). doi:10.5281/ZENODO.887916.
6. Pedregosa, F. *et al.* Scikit-learn: Machine Learning in Python. *Journal of Machine Learning Research* **12**, 2825–2830 (2011).
7. Sainburg, T., McInnes, L. & Gentner, T. Q. Parametric UMAP Embeddings for Representation and Semisupervised Learning. *Neural Comput* **33**, 2881–2907 (2021).
8. Wolf, F. A., Angerer, P. & Theis, F. J. SCANPY: large-scale single-cell gene expression data analysis. *Genome Biology* **19**, 1–5 (2018).
9. Traag, V. A., Waltman, L. & van Eck, N. J. From Louvain to Leiden: guaranteeing well-connected communities. *Scientific Reports* **9**, 1–12 (2019).
10. Lötsch, J., Kringel, D. & Ultsch, A. Revisiting Fold-Change Calculation: Preference for Median or Geometric Mean over Arithmetic Mean-Based Methods. *Biomedicines* **12**, (2024).
11. Virtanen, P. *et al.* SciPy 1.0: fundamental algorithms for scientific computing in Python. *Nat Methods* **17**, 261–272 (2020).
12. Safir, F. *et al.* Combining Acoustic Bioprinting with AI-Assisted Raman Spectroscopy for High-Throughput Identification of Bacteria in Blood. *Nano Lett* **23**, 2065–2073 (2023).
13. Zheng, G. X. Y. *et al.* Massively parallel digital transcriptional profiling of single cells. *Nature*

Communications **8**, 1–12 (2017).

14. Virshup, I., Rybakov, S., Theis, F. J., Angerer, P. & Alexander Wolf, F. anndata: Access and store annotated data matrices. *Journal of Open Source Software* **9**, 4371 (2024).
15. Gayoso, A. & Shor, J. JonathanShor/DoubletDetection: doubletdetection v4.3.0.post1. doi:10.5281/zenodo.14827937.
16. Lopez, R., Regier, J., Cole, M. B., Jordan, M. I. & Yosef, N. Deep generative modeling for single-cell transcriptomics. *Nature Methods* **15**, 1053–1058 (2018).
17. Virshup, I. *et al.* The scverse project provides a computational ecosystem for single-cell omics data analysis. *Nature Biotechnology* **41**, 604–606 (2023).
18. Gayoso, A. *et al.* A Python library for probabilistic analysis of single-cell omics data. *Nature Biotechnology* **40**, 163–166 (2022).
19. Xu, C. *et al.* Probabilistic harmonization and annotation of single-cell transcriptomics data with deep generative models. *Molecular Systems Biology* (2021) doi:10.15252/msb.20209620.
20. Seabold, S. & Perktold, J. Statsmodels: Econometric and Statistical Modeling with Python. *SciPy 2010* (2010) doi:10.25080/Majora-92bf1922-011.
21. Potcoava, M. C., Futia, G. L., Gibson, E. A. & Schlaepfer, I. R. Raman Microscopy Techniques to Study Lipid Droplet Composition in Cancer Cells. *Methods Mol Biol* **2413**, 193–209 (2022).
22. Chaturvedi, D. *et al.* Different Phases of Breast Cancer Cells: Raman Study of Immortalized, Transformed, and Invasive Cells. *Biosensors (Basel)* **6**, (2016).
23. Ding, H. *et al.* analysis of mucosal lipids reveals histological disease activity in ulcerative colitis using endoscope-coupled Raman spectroscopy. *Biomed Opt Express* **8**, 3426–3439 (2017).
24. Kuhar, N., Sil, S., Verma, T. & Umapathy, S. Challenges in application of Raman spectroscopy to biology and materials. *RSC Adv* **8**, 25888–25908 (2018).

25. Stiebing, C. *et al.* Raman imaging of macrophages incubated with triglyceride-enriched oxLDL visualizes translocation of lipids between endocytic vesicles and lipid droplets. *J Lipid Res* **58**, 876–883 (2017).

26. Lee, H. J. *et al.* Multimodal Metabolic Imaging Reveals Pigment Reduction and Lipid Accumulation in Metastatic Melanoma. *BME Front* **2021**, 9860123 (2021).

27. Kanno, N. *et al.* Machine learning-assisted single-cell Raman fingerprinting for and nondestructive classification of prokaryotes. *iScience* **24**, 102975 (2021).

28. Reinmuth-Selzle, K. *et al.* Determination of the protein content of complex samples by aromatic amino acid analysis, liquid chromatography-UV absorbance, and colorimetry. *Anal Bioanal Chem* **414**, 4457–4470 (2022).

29. Xie, Y., Zhang, D., Jarori, G. K., Davisson, V. J. & Ben-Amotz, D. The Raman detection of peptide tyrosine phosphorylation. *Anal Biochem* **332**, 116–121 (2004).

30. Siamwiza, M. N. *et al.* Interpretation of the doublet at 850 and 830 cm-1 in the Raman spectra of tyrosyl residues in proteins and certain model compounds. *Biochemistry* **14**, 4870–4876 (1975).

31. Abramczyk, H. *et al.* Aberrant Protein Phosphorylation in Cancer by Using Raman Biomarkers. *Cancers (Basel)* **11**, (2019).

32. Flower, C. T. *et al.* Signaling and transcriptional dynamics underlying early adaptation to oncogenic BRAF inhibition. *Cell Syst* **16**, 101239 (2025).

33. Smith, L. K. *et al.* Adaptive translational reprogramming of metabolism limits the response to targeted therapy in BRAF melanoma. *Nat Commun* **13**, 1100 (2022).

34. Gerosa, L. *et al.* Receptor-Driven ERK Pulses Reconfigure MAPK Signaling and Enable Persistence of Drug-Adapted BRAF-Mutant Melanoma Cells. *Cell Syst* **11**, 478–494.e9 (2020).

35. Ravindran Menon, D. *et al.* A stress-induced early innate response causes multidrug tolerance in melanoma. *Oncogene* **34**, 4448–4459 (2015).

36. Pazarentzos, E. & Bivona, T. G. Adaptive stress signaling in targeted cancer therapy resistance. *Oncogene* **34**, 5599–5606 (2015).
37. Brauchle, E., Thude, S., Brucker, S. Y. & Schenke-Layland, K. Cell death stages in single apoptotic and necrotic cells monitored by Raman microspectroscopy. *Sci Rep* **4**, 4698 (2014).
38. Ong, Y. H., Lim, M. & Liu, Q. Comparison of principal component analysis and biochemical component analysis in Raman spectroscopy for the discrimination of apoptosis and necrosis in K562 leukemia cells. *Opt Express* **20**, 22158–22171 (2012).
39. Owen, C. A. *et al.* In vitro toxicology evaluation of pharmaceuticals using Raman micro-spectroscopy. *J Cell Biochem* **99**, 178–186 (2006).
40. Notingher, I. Raman Spectroscopy Cell-based Biosensors. *Sensors (Basel, Switzerland)* **7**, 1343 (2007).
41. Chan, J. W., Taylor, D. S. & Thompson, D. L. The effect of cell fixation on the discrimination of normal and leukemia cells with laser tweezers Raman spectroscopy. *Biopolymers* **91**, 132–139 (2009).
42. Yao, J., Wang, L., Li, C., Zhang, C. & Wang, L. V. Photoimprint photoacoustic microscopy for three-dimensional label-free subdiffraction imaging. *Phys Rev Lett* **112**, 014302 (2014).

Supplementary Tables

Mouse cell line viability				
Cell line	Bem	Cabo	Dabra	Nivo
YUMM1.7	68.3	74.4	77.0	71.4
YUMMER1.7	57.1	82.8	89.2	81.1

Human cell line viability				
Cell line	Bem	Cabo	Dabra	Nivo
A375	86.1	76.1	66.1	82.7
SK-MEL-24	61.3	64.7	84.6	78.6
SK-MEL-30	70.5	79.8	78.6	80.1

Patient derived tumor line cell viability						
Sample ID	Bem	Cabo	Dabra	Nivo	Nivo + Rela	Clinical
PAT-52	62.9	82.6	91.6	40.5	80.4	-
PAT-54	70.4	72.4	89.6	45.6	85.4	-
PAT-63	74.1	80.4	82.4	78.6	24.6	NR (Nivo); R (Nivo + Rela)
PAT-73	92.0	71.2	92.1	86.7	90.6	NR (Nivo)
PAT-93	52.4	69.4	82.4	66.1	90.4	NR (Dabra); NR (Nivo)
PAT-68	83.4	85.2	74.8	86.4	27.4	NR (Nivo)
PAT-03	62.7	65.4	81.9	66.1	-	-
PAT-81	90.1	78.4	81.9	91.7	92.7	NR (Dabra); NR (Nivo)
PAT-77	86.4	76.4	74.0	82.9	86.6	NR (Nivo); NR (Nivo + Rela)
PAT-03.P2	72.7	73.4	80.4	70.1	-	-
PAT-73.P2	89.0	61.2	81.1	85.7	89.0	-
PAT-73.P3	71.0	77.2	89.1	90.7	91.1	-

Supplementary Table 1. Cell viability data of mice and human melanoma samples. In vitro cell viability assessments for the first passages were determined after 24 hour incubation of inhibitors with concentrations described in Table 3 to observe cell response for mice samples and human samples. Additionally, cell viability was determined for later passages on select patients of interest (PAT-73, PAT-03). Viability values correspond to relative percentage to untreated control samples. For the patient resistance determination model, resistant or sensitive labels were assigned based on cell viability inference or clinical outcomes in select cases. No clinical outcome labels (R for responder and NR for nonresponder) conflicted with cell viability observations.

Inhibitor concentrations (μM)					
**Sample**	**Bem**	**Cabo**	**Dabra**	**Nivo**	**Nivo + Rela**
YUMM1.7	1	7	1	5	-
YUMMER1.7	1	8.5	3.5	8	-
A375	8	7.5	5	5	-
SK-MEL-24	8	9.5	10	10	-
SK-MEL-30	8.5	8.5	1	8	-
Patient derived tumor lines	8	9.5	5	6	6 + 7

Supplementary Table 2. Inhibitor concentrations used in each of our datasets.

Cell response cell count					
Cell line	Control	Bem	Cabo	Dabra	Nivo
YUMM1.7	886	1025	1146	1081	1004
YUMMER1.7	1078	961	958	838	960
A375	1291	3103	2884	1267	1303
SK-MEL-24	1299	1473	1423	1422	1430
SK-MEL-30	3327	1506	1366	1680	1380
PAT-52	1584	430	336	505	455
PAT-73	2131	406	566	761	593

Patient control cell count	
Sample ID	Count
PAT-52	1581
PAT-54	1609
PAT-63	557
PAT-68	284
PAT-73	2131
PAT-77	96
PAT-81	199
PAT-93	657
PAT-03	1512
PAT-03.P2	1591
PAT-73.P2	985
PAT-73.P3	1388

Patient control cell count	
Sample ID	Count
PAT-52.P2	1391
PAT-54.P2	1386
PAT-68.P2	1381
PAT-63.P2	1329

Supplementary Table 3. Spectra count acquired for each sample utilized in our response or determination dataset. The first table represents spectra counts utilized in observing cell response to inhibitor presence. The second table represents spectra counts utilized in our patient prediction model analysis. The third table represents spectra counts from additional passages of select patients used in understanding passage shift.

Supplementary Table 4. Raman band assignments associated with tumor immune microenvironment cells.

Raman band (cm^{-1})	Band assignment	Biological group	Reference
644-645	C-C twisting in Tyrosine	Proteins	[1–4]
654	Tyrosine, G	Proteins, Nucleic acid	[5]
667	T, G	Nucleic acids	[2,6]
708	C	Nucleic acids	[7,8]
731	A (ring breathing)	Nucleic acids	[2]
745-750	Ring breathing vibrations of nucleic acids<br>Tryptophan	Nucleic acids<br>Proteins	[9]
779-784	C, U (ring breathing), PO2- backbone stretch	Nucleic acids	[2,10]
805	PO2- symmetric stretch	Nucleic acids	[11]
826	Tyrosine (ring breathing)	Proteins	[12]
829	Ring breathing	Proteins	[13,14]
833-836	PO2- symmetric stretch	Nucleic acids	[12]
841-843	Polysaccharide, Glucose	Carbohydrates	[6]
845-851	C-C stretch in Tyrosine and Valine	Proteins	[4,13,15]
876	Tryptophan	Proteins	[14,16]
883	CH2 rocking	Proteins	[6]
888	Tyrosine	Proteins	[17]
904	Tyrosine<br>Glycogen	Proteins<br>Carbohydrates	[18,19]
912	Glucose	Carbohydrates	[6,20]
917	C-C stretch	Proteins	[20]
924	Proline ring	Protein	[11]
927-940	C-C skeletal stretch	Proteins	[6,12,21]
955	C-C stretch	Proteins	[3]
958-959	CH3 deformations	Lipids, Proteins	[22]
968	Phosphatidylcholine	Lipids	[12]
998-1003	C-C aromatic symmetric ring breathing of phenylalanine	Proteins	[23–25]

1021	Glycogen	Carbohydrates	[6]
1030-1032	C-N in-plane bending of phenylalanine	Proteins	[12]
1080-1082	C-C<br>PO2 symmetric stretch	Lipids<br>Nucleic acids	[26,27]
1126-1129	C–C stretch<br>C–N stretch	Lipids<br>Proteins	[1,25]
1156-1158	C-C stretch, C-N stretch	Beta-carotene<br>Proteins	[4,27–29]
1173-1174	Tyrosine, Phenylalanine	Proteins	[2]
1205-1207	C-C6H5 stretch in phenylalanine and tryptophan<br>Tyrosine	Proteins	[4,12,30]
1220	Amide III	Proteins	[31]
1225	Amide III	Proteins	[6]
1241	T, Amide III	Nucleic acids<br>Proteins	[2]
1250-1252	Amide III	Proteins	[12]
1270	C-H bending	Lipids	[32]
1274	Amide III	Proteins	[33]
1303-1306	A (ring mode),<br>C-H deformation<br>CH2 twist	Nucleic acids<br>Proteins<br>Lipids	[2,12,34,35]
1308-1310	CH2 deformation, CH3CH2 twisting	Lipids	[9,30]
1312	CH3CH2 twisting	Proteins, Lipids	[31]
1336	CH3, CH2 wagging, C-H deformation, C-C stretch	Proteins, Lipids,<br>Carbohydrates	[15,36]
1337	A, G (ring mode)	Nucleic acids	[29]
1352	G<br>Tryptophan	Nucleic acids<br>Proteins	[37]
1361	Tryptophan	Proteins	[20]
1382	CH3 symmetric deformation<br>Ring breathing modes of the nucleic acids	Lipids<br>Nucleic acids	[38]
1385	C-C linear stretch<br>CH3 symmetric deformation	Melanin<br>Lipids	[39–42]
1400	U, A	Nucleic acids	[8]
1410-1413	CH deformation	Proteins<br>Lipids	[43]

1419-1420	CH2 scissoring<br>A, G	Lipids<br>Nucleic acids	[12]
1433-1440	CH2 deformation, C=C stretch unsaturated fatty acids	Lipids	[9,10]
1445-1447	CH2 bend<br>CH2 deformation	Proteins<br>Lipids	[2,10,25]
1457-1460	CH2 deformation, CH deformation	Proteins	[2,12]
1470	G, A	Nucleic acid	[44]
1482	G, A (ring mode)	Nucleic acids	[12]
1497	C-NH2 scissoring	Nucleic acids	[45]
1518	C=C stretch	Carotenoid	[6]
1526-1527	C=C	Carotenoids	[2]
1553	Tryptophan	Proteins	[12]
1562	C-N and N-H vibrations<br>Tryptophan<br>A, G ring breathing	Proteins<br>Nucleic acids	[38]
1575	C-C ring stretch	Nucleic acids	[12,15]
1580	Aromatic ring in-plane stretch	Melanin	[39]
1582-1588	A,G<br>C=C olefinic stretch<br>Tyrosine	Nucleic acids<br>Proteins	[6,9,13,27,39–41,46]
1591-1593	C=C in-plane bending mode in Phenylalanine	Proteins	[42]
1606	C=C stretch in Phenylalanine	Proteins	[47]
1610	aromatic ring stretch in Tyrosine	Proteins	[4]
1624	C=C bend in tryptophan	Proteins	[45]
1636	Amide I	Proteins	[6]
1652-1658	C=C stretch, Amide I	Lipids, Proteins	[9,29,42]
1660-1666	Amide I	Proteins	[12,30]
1672	Amide I	Proteins	[15,33]
1681-1684	U (C=O stretch)	Nucleic acids	[12]
1692	Amide I	Proteins	[7]
1735-1775	C=O stretch	Lipids	[27,42,48]
1791-1797		Unassigned	[49]

1. Chaudhary, N., Nguyen, T. N. Q., Cullen, D., Meade, A. D. & Wynne, C. Discrimination of immune cell activation using Raman micro-spectroscopy in an in-vitro & ex-vivo model. *Spectrochim Acta A Mol Biomol Spectrosc* **248**, 119118 (2021).
2. Brauchle, E. *et al.* Raman spectroscopy as an analytical tool for melanoma research. *Clin Exp Dermatol* **39**, 636–645 (2014).
3. Qiu, X. *et al.* Raman spectroscopy combined with deep learning for rapid detection of melanoma at the single cell level. *Spectrochim Acta A Mol Biomol Spectrosc* **286**, 122029 (2023).
4. Xie, Y., Zhang, D., Jarori, G. K., Davisson, V. J. & Ben-Amotz, D. The Raman detection of peptide tyrosine phosphorylation. *Anal Biochem* **332**, 116–121 (2004).
5. Zhang, P. *et al.* Label-Free Exosomal Detection and Classification in Rapid Discriminating Different Cancer Types Based on Specific Raman Phenotypes and Multivariate Statistical Analysis. *Molecules* **24**, (2019).
6. Movasaghi, Z., Rehman, S. & Rehman, I. U. Raman Spectroscopy of Biological Tissues. *Applied Spectroscopy Reviews* **42**, 493–541 (2007).
7. Wang, J. *et al.* Raman spectroscopic study of cervical precancerous lesions and cervical cancer. *Lasers Med Sci* **36**, 1855–1864 (2021).
8. Bankapur, A., Zachariah, E., Chidangil, S., Valiathan, M. & Mathur, D. Raman tweezers spectroscopy of live, single red and white blood cells. *PLoS One* **5**, e10427 (2010).
9. Feuerer, N. *et al.* Lipidome profiling with Raman microspectroscopy identifies macrophage response to surface topographies of implant materials. *Proceedings of the National Academy of Sciences* **118**, e2113694118 (2021).
10. Naumann, M. *et al.* Label-Free Characterization of Macrophage Polarization Using Raman Spectroscopy. *International Journal of Molecular Sciences* **24**, 824 (2023).
11. Mahadevan-Jansen, A. & Richards-Kortum, R. R. Raman spectroscopy for the detection of cancers and precancers. *J Biomed Opt* **1**, 31–70 (1996).
12. Terentis, A. C., Fox, S. A., Friedman, S. J. & Spencer, E. S. Confocal Raman microspectroscopy discriminates live human metastatic melanoma and skin fibroblast cells. *Journal of Raman Spectroscopy* **44**, 1205–1216 (2013).

13. Abramczyk, H. *et al.* Aberrant Protein Phosphorylation in Cancer by Using Raman Biomarkers. *Cancers (Basel)* **11**, (2019).
14. Kuhar, N., Sil, S., Verma, T. & Umapathy, S. Challenges in application of Raman spectroscopy to biology and materials. *RSC Adv* **8**, 25888–25908 (2018).
15. Zhou, Q.-Q. *et al.* Rapid visualization of PD-L1 expression level in glioblastoma immune microenvironment via machine learning cascade-based Raman histopathology. *J Adv Res* **65**, 257–271 (2024).
16. Chaturvedi, D. *et al.* Different Phases of Breast Cancer Cells: Raman Study of Immortalized, Transformed, and Invasive Cells. *Biosensors (Basel)* **6**, (2016).
17. Chen, X., Li, X., Yang, H., Xie, J. & Liu, A. Diagnosis and staging of diffuse large B-cell lymphoma using label-free surface-enhanced Raman spectroscopy. *Spectrochim Acta A Mol Biomol Spectrosc* **267**, 120571 (2022).
18. Lee, S. *et al.* Early-stage diagnosis of bladder cancer using surface-enhanced Raman spectroscopy combined with machine learning algorithms in a rat model. *Biosens Bioelectron* **246**, 115915 (2024).
19. Mert, S., Özbek, E., Ötünçtemur, A. & Çulha, M. Kidney tumor staging using surface-enhanced Raman scattering. *J Biomed Opt* **20**, 047002 (2015).
20. Chang, M. *et al.* RaT: Raman Transformer for highly accurate melanoma detection with critical features visualization. *Spectrochimica Acta Part A: Molecular and Biomolecular Spectroscopy* **305**, 123475 (2024).
21. Delrue, C., Speeckaert, R., Oyaert, M., De Bruyne, S. & Speeckaert, M. M. From Vibrations to Visions: Raman Spectroscopy's Impact on Skin Cancer Diagnostics. *J Clin Med* **12**, (2023).
22. Nijssen, A. *et al.* Discriminating basal cell carcinoma from its surrounding tissue by Raman spectroscopy. *J Invest Dermatol* **119**, 64–69 (2002).
23. You, C., Yi, J.-Y., Hsu, T.-W. & Huang, S.-L. Integration of cellular-resolution optical coherence tomography and Raman spectroscopy for discrimination of skin cancer cells with machine learning. *JBO* **28**, 096005 (2023).
24. Ribeiro, A. R. B. *et al.* Application of Raman spectroscopy for characterization of the functional polarization of macrophages into M1 and M2 cells. *Spectrochim Acta A Mol Biomol Spectrosc* **265**,

120328 (2022).

25. Wen, J. *et al.* Detection and Classification of Multi-Type Cells by Using Confocal Raman Spectroscopy. *Front. Chem.* **9**, 641670 (2021).

26. Yang, C. *et al.* The Identification of Breast Cancer Subtypes by Raman Spectroscopy Integrated With Machine Learning Algorithms: Analyzing the Influence of Baseline. *Journal of Raman Spectroscopy* (2025) doi:10.1002/jrs.6799.

27. Rimskaya, E. *et al.* Multi-Wavelength Raman Differentiation of Malignant Skin Neoplasms. *International Journal of Molecular Sciences* **25**, 7422 (2024).

28. Huang, L. *et al.* Rapid, label-free histopathological diagnosis of liver cancer based on Raman spectroscopy and deep learning. *Nature Communications* **14**, 1–14 (2023).

29. Feng, X. *et al.* Raman active components of skin cancer. *Biomed Opt Express* **8**, 2835–2850 (2017).

30. Xie, J., Wei, Y., Liang, X., Ma, C. & Su, X. In situ single-cell spontaneous Raman spectroscopy differentiates tumor-associated macrophages. *Sensors and Actuators B: Chemical* **417**, 136053 (2024).

31. Lazaro-Pacheco, D., Shaaban, A. M., Rehman, S. & Rehman, I. Raman spectroscopy of breast cancer. *Applied Spectroscopy Reviews* (2020) doi:10.1080/05704928.2019.1601105.

32. Ding, H. *et al.* analysis of mucosal lipids reveals histological disease activity in ulcerative colitis using endoscope-coupled Raman spectroscopy. *Biomed Opt Express* **8**, 3426–3439 (2017).

33. Gniadecka, M. *et al.* Melanoma diagnosis by Raman spectroscopy and neural networks: structure alterations in proteins and lipids in intact cancer tissue. *J Invest Dermatol* **122**, 443–449 (2004).

34. Puppels, G. J., Garritsen, H. S., Segers-Nolten, G. M., de Mul, F. F. & Greve, J. Raman microspectroscopic approach to the study of human granulocytes. *Biophys J* **60**, 1046–1056 (1991).

35. Zhang, X. *et al.* Investigation on the Cancer Invasion and Metastasis of Skin Squamous Cell Carcinoma by Raman Spectroscopy. *Molecules* **24**, 2059 (2019).

36. Danciu, C. *et al.* A characterization of four B16 murine melanoma cell sublines molecular fingerprint and proliferation behavior. *Cancer Cell Int* **13**, 75 (2013).

37. Aguilar-Hernández, I. *et al.* Discrimination of radiosensitive and radioresistant murine lymphoma cells by Raman spectroscopy and SERS. *Biomed Opt Express* **11**, 388–405 (2020).

38. Silveira, L., Jr, Silveira, F. L., Bodanese, B., Zângaro, R. A. & Pacheco, M. T. T. Discriminating model for diagnosis of basal cell carcinoma and melanoma in vitro based on the Raman spectra of selected biochemicals. *J Biomed Opt* **17**, 077003 (2012).

39. Huang, Z. *et al.* Raman spectroscopy of in vivo cutaneous melanin. *JBO* **9**, 1198–1205 (2004).

40. Yakimov, B. P. *et al.* Melanin distribution from the dermal–epidermal junction to the stratum corneum: non-invasive in vivo assessment by fluorescence and Raman microspectroscopy. *Scientific Reports* **10**, 1–13 (2020).

41. Lim, L. *et al.* Clinical study of noninvasive in vivo melanoma and nonmelanoma skin cancers using multimodal spectral diagnosis. *J Biomed Opt* **19**, 117003 (2014).

42. Paidi, S. K. *et al.* Raman Spectroscopy and Machine Learning Reveals Early Tumor Microenvironmental Changes Induced by Immunotherapy. *Cancer Res* **81**, 5745–5755 (2021).

43. Kourkoumelis, N. *et al.* Advances in the in Vivo Raman Spectroscopy of Malignant Skin Tumors Using Portable Instrumentation. *Int J Mol Sci* **16**, 14554–14570 (2015).

44. Short, K. W., Carpenter, S., Freyer, J. P. & Mourant, J. R. Raman spectroscopy detects biochemical changes due to proliferation in mammalian cell cultures. *Biophys J* **88**, 4274–4288 (2005).

45. Pezzotti, G. Raman spectroscopy in cell biology and microbiology. *Journal of Raman Spectroscopy* **52**, 2348–2443 (2021).

46. Borek-Dorosz, A. *et al.* Raman-based spectrophenotyping of the most important cells of the immune system. *J Adv Res* **41**, 191–203 (2022).

47. Sigle, M. *et al.* Translating genomic tools to Raman spectroscopy analysis enables high-dimensional tissue characterization on molecular resolution. *Nature Communications* **14**, 1–16 (2023).

48. Elumalai, S., Managó, S. & De Luca, A. C. Raman Microscopy: Progress in Research on Cancer Cell Sensing. *Sensors* **20**, 5525 (2020).

49. Grajales Lopera, D. O. *et al.* Image-guided Raman spectroscopy navigation system to improve transperineal prostate cancer detection. Part 2: in-vivo tumor-targeting using a classification model combining spectral and MRI-radiomics features. *J Biomed Opt* **27**, (2022).
